# Rapid X-Ray-Based 3-D Finite Element Modeling of Medial Knee Joint Cartilage Biomechanics During Walking

**DOI:** 10.1007/s10439-022-02941-0

**Published:** 2022-03-09

**Authors:** Sana Jahangir, Ali Mohammadi, Mika E. Mononen, Jukka Hirvasniemi, Juha-Sampo Suomalainen, Simo Saarakkala, Rami K. Korhonen, Petri Tanska

**Affiliations:** 1 Department of Applied Physics, University of Eastern Finland, POB 1627, 70211 Kuopio, Finland; 2 Department of Radiology & Nuclear Medicine, Erasmus MC University Medical Center, Rotterdam, The Netherlands; 3 Diagnostic Imaging Center, Kuopio University Hospital, Kuopio, Finland; 4 Research Unit of Medical Imaging, Physics and Technology, Faculty of Medicine, University of Oulu, Oulu, Finland; 5 Department of Diagnostic Radiology, Oulu University Hospital, Oulu, Finland

**Keywords:** Finite element modeling, Atlas-based modeling, Articular cartilage, Planar radiography, Magnetic resonance imaging, Knee osteoarthritis

## Abstract

Finite element (FE) modeling is becoming an increasingly popular method for analyzing knee joint mechanics and biomechanical mechanisms leading to osteoarthritis (OA). The most common and widely available imaging method for knee OA diagnostics is planar X-ray imaging, while more sophisticated imaging methods, e.g., magnetic resonance imaging (MRI) and computed tomography (CT), are seldom used. Hence, the capability to produce accurate biomechanical knee joint models directly from X-ray imaging would bring FE modeling closer to clinical use. Here, we extend our atlas-based framework by generating FE knee models from X-ray images (*N* = 28). Based on measured anatomical landmarks from X-ray and MRI, knee joint templates were selected from the atlas library. The cartilage stresses and strains of the X-ray-based model were then compared with the MRI-based model during the stance phase of the gait. The biomechanical responses were statistically not different between MRI- vs. X-ray-based models when the template obtained from X-ray imaging was the same as the MRI template. However, if this was not the case, the peak values of biomechanical responses were statistically different between X-ray and MRI models. The developed X-ray-based framework may pave the way for a clinically feasible approach for knee joint FE modeling.

**Supplementary Information:**

The online version contains supplementary material available at 10.1007/s10439-022-02941-0.

## Introduction

Osteoarthritis (OA) is one of the major causes of disability in elderly people.^17^ Computational finite element (FE) models have been utilized to quantitatively estimate biomechanical factors applied to the soft tissue of the knee joint during different loading conditions.^1,2,13^ There is considerable evidence that local tissue stresses and strains are one of the driving factors for knee OA.^3,11^ In recent years, these simulated biomechanical responses have been utilized in predictive FE models to predict biomechanically-driven progression of knee OA based on clinical imaging data.^10,33^ In those studies, subject-specific 3-D joint geometries of the models have been based on computed tomography (CT) or magnetic resonance imaging (MRI). The potential of X-ray imaging under a similar workflow has not been evaluated so far. As in primary healthcare, planar radiographs are the primary imaging modality for knee OA diagnostics,^16^ their compatibility in the modeling workflow would be a valuable asset. The capability to generate 3-D knee joint models from 2-D radiographs could also increase the scalability of the FE modeling to large patient groups.

Various methods for the 3-D reconstruction of shape and geometry from X-ray images have been developed.^12,19^ These methods include conventional image segmentation methods, e.g., contour matching, edge detection and statistical shape modeling.^4,9,12,19,39,48^ Methods that include only radiographs as input images are challenging to use in soft tissue segmentation due to very limited soft tissue contrast. Moreover, region-based algorithms are prone to noise and over-segmentation of the X-ray images.^45^ Statistical shape model (SSM)-based geometry generation has been shown to produce a good accuracy in generation of knee geometries but it requires sufficiently high-quality and large datasets to capture all possible shape variations.^12^ Though there are several publicly available clinical datasets for digital health research,^21^ the available datasets are often noisy, incomplete and contain artifacts as clinical imaging is tailored to physicians’ needs and to minimize harm to patients.^27^

In any clinical application, FE model generation must be fast. Various methods have been applied to reduce model generation times utilizing different semiautomatic and automatic segmentation and meshing techniques.^32,37,43^ However, issues related to the image quality and artifacts, as well as the substantial amount of manual labor required for the generation and simulation of FE knee joint models, present challenges when using these techniques. An approach introduced by Rodriguez-Vila *et al*.^37^ for the rapid generation of FE meshes is quite promising but has not been tested for a large group of subjects. Recently, we developed an atlas-based FE knee joint modeling workflow utilizing knee CT images for rapid knee FE model construction and simulation without cartilage segmentation.^32^ We observed that CT-based knee models produce similar mechanical responses in knee joint cartilage as MRI-based models.^30,32^ This was an important step and introduces the possibility of using native X-ray imaging, where only bones are visible, for FE model generation.

Thus, the key purpose of this study is to investigate whether an automated knee joint FE modeling approach based on knee radiographs can simulate mechanical responses of knee joint cartilage with similar accuracy as our previously verified MRI-based knee joint FE modeling approach.^25,32^ We hypothesized that the previously presented approach can generate 3-D knee joint FE models based on 2-D knee radiographs and that these models can simulate similar cartilage mechanical responses when compared to the models generated from MR images.

## Materials and Methods

### Subject Information

Previously collected data from 28 patients with ages between 48 and 65 years were used in this study.^18^ Written consent of each patient was taken. The data was collected in compliance with the Declaration of Helsinki, and the ethical permission was granted by the Ethical Committee of Northern Ostrobothnia Hospital District, Oulu University Hospital (number 7/2016).^18^

### Image Acquisition

The magnetic resonance (MR) and X-ray images of the knee joint of each patient were acquired using a clinical 3 T MRI scanner (Siemens Skyra, Siemens Healthcare) and X-ray device (DigitalDiagnost, Philips Medical Systems).^18^ Bilateral posterior–anterior (i.e., front part of the knee was towards the detector) and lateral (sagittal) knee radiographs were acquired according to normal clinical routine in a weight-bearing and semi-flexed position (10° X-ray beam angle, 60 kVp, automatic exposure, source–detector distance 153 cm, pixel size 0.148 mm × 0.148 mm).^18^ The posterior–anterior X-ray images were taken using the SynaFlexer™ X-ray positioning frame (Synarc, Inc., San Francisco, USA) while no knee positioning frame was used for the lateral images. Therefore, there was some variation in the flexion of the knee in the lateral images of X rays. The rotation of the knee was minimized so that the femoral condyles would be superimposed. MRI scans of knee joints of all subjects were also taken on the same day using a sagittal T2-weighted dual-echo steady-state sequence [echo time (*T*_E_) 5 ms, echo train length (ETL) 2, repetition time (*T*_R_) 14.1 ms, slice thickness 0.6 mm, pixel size: 0.6 mm × 0.6 mm]. In our study, the right knees of the patients were analyzed. An experienced radiologist scored these radiographs using the Kellgren–Lawrence grading, and patients had no evidence of clinical cartilage erosion (i.e., 14 patients with KL-0 and 14 patients with KL-1 indicating no evidence of joint space narrowing).^18^

**Figure 1:**
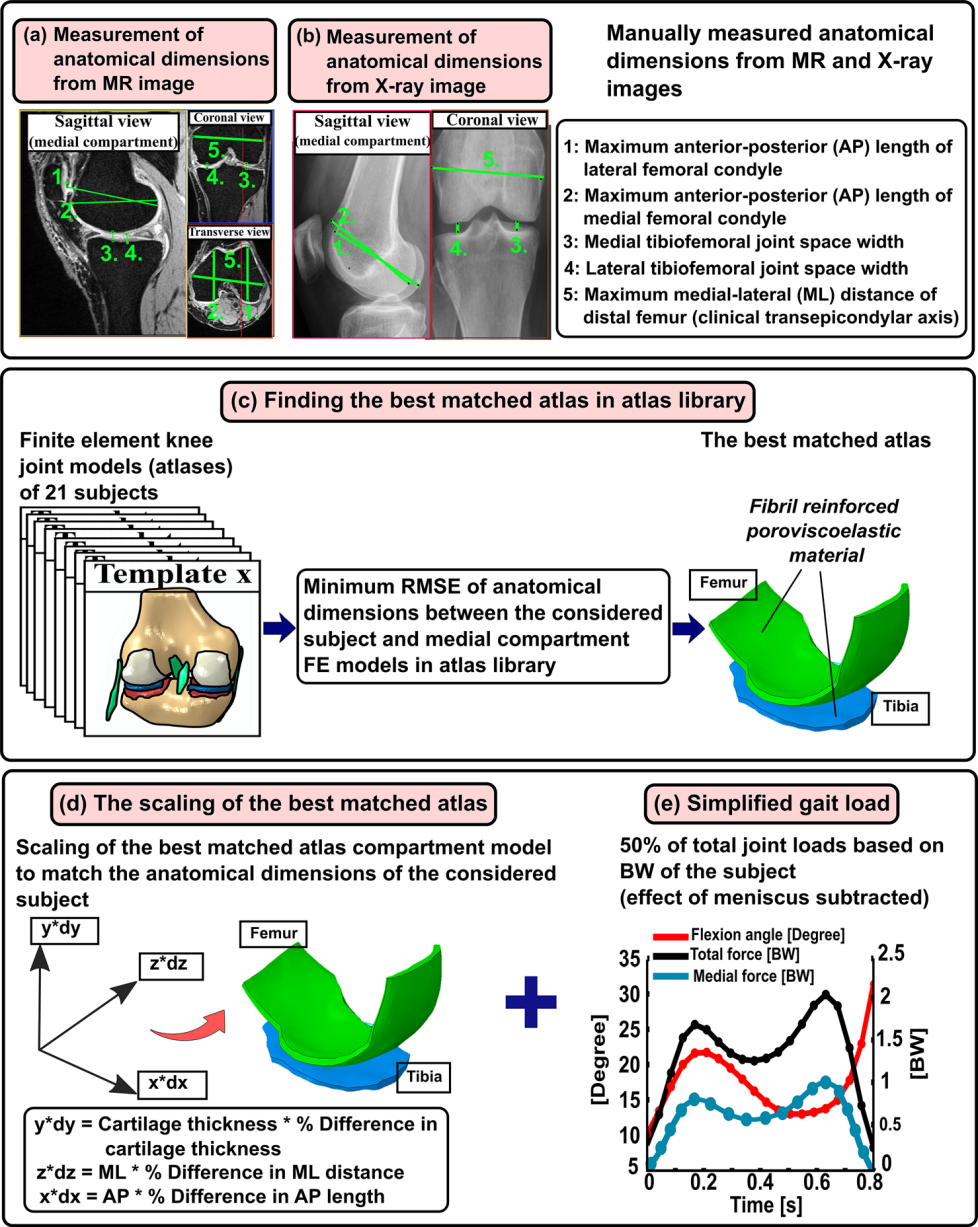
Workflow of the presented study. The first row shows the manual labor required for model generation by the atlas-based FE knee joint model framework. (**a** and **b**) Five anatomical dimensions were measured from MR images of each subject (*N* = 28) considered in this study to generate MRI-based knee joint atlas models. Anatomical landmarks were also measured similarly from clinical MR images (OAI database) for each knee joint geometry (*N* = 21) in the atlas library, and this information was linked with a corresponding medial compartment atlas model. (**c**) Finding best-matched atlas from atlas library based on minimum root mean square error (RMSE) of anatomical landmarks between considered subject and all 21 atlases in atlas library. (**d**) The optimal atlas compartment model was scaled to match anatomical landmarks of the considered subject by multiplication of its nodal coordinate values with percentage difference in AP, ML and tibiofemoral cartilage thickness directions (d*x*, d*y* and d*z*) between the subject data and best-matched atlas. (**e**) The biomechanical response of the scaled atlas FE compartment model was simulated using the physiologically relevant gait loading (50% of total joint loads obtained from whole knee joint simulations were assumed to occur in the medial compartment) based on body weight (BW) of the subject. The contribution of medial meniscus was considered in the compartment model by subtracting the simplified gait loading by the average contribution of medial meniscus.

The workflow of the atlas-based method is shown in Fig. 1.

### Generation of Knee Joint FE Model Atlas Database

The details of the knee joint FE model atlas database generation are described in detail in our previous studies.^32^ Briefly, 3D knee joint geometries (*N* = 21) from the MR images obtained from the OAI database (https://nda.nih.gov/oai/) were created in earlier studies^25,32^ using Mimics v15.01 (Materialise, Leuven, Belgium). Final geometries were imported in Abaqus software (Abaqus/CAE v6.18, Dassault Systèmes, Providence, RI, USA), in which the FE models were constructed. Subsequently, to enhance the computational convergence and efficiency, only medial compartment FE models were considered in further simulations. Finally, anatomical landmarks were measured from clinical MR images (OAI database) for each knee joint geometry using our in-house build script in MATLAB, and this information was linked with a corresponding medial compartment model. The five measured anatomical dimensions included the maximum anterior–posterior (AP) distance of the medial and lateral condyles of distal femur, tibiofemoral joint space width (JSW) of the medial and lateral compartments, and lastly, the maximum medial–lateral (ML) width of distal femur. The AP dimensions from MR images were determined as the maximum AP distance of ellipsoidal shaped medial and lateral condyles (major axis of the ellipsoid). Tibiofemoral JSWs were measured at the central location of the tibiofemoral contact region in medial and lateral compartments. ML width was the maximum distance between the medial and lateral epicondyles, i.e., clinical transepicondylar axis (Fig. 1a).

### Generation of X-Ray-Based Knee Joint FE Models

To develop X-ray- and MRI-based knee joint FE models for the study subjects (*N* = 28), the previously described atlas-based approach was utilized (Figs. 1a and 1b).^32^ In this approach, the aforementioned anatomical dimensions of the distal femur (AP and ML) and tibiofemoral JSWs were measured. To mimic these measurements in X-ray images as taken from MR images, both AP dimensions were determined from the sagittal plane, whereas JSWs and ML dimensions were obtained from the coronal plane (Fig. 1b). ImageJ^38^ (Rasband, W.S., National Institutes of Health, Maryland, USA) was used to measure these dimensions. Although the sagittal images of X rays were differently oriented than MRIs, the approach of taking the maximum AP length of ellipsoidal shaped medial and lateral condyles was kept the same in both MR and X-ray images. To account for slight image magnification in radiographs, each posterior–anterior and lateral X-ray image contained a calibration disc (diameter = 30 mm) placed in a precise position in the field of view and the disc was used to calibrate the image dimensions. Afterward, these measurements were repeated for the MR images of the same knees. Anatomical dimensions were measured three times from both X-ray and MR images of each patient by the same rater to evaluate the intra-rater reliability^14^ of the measurements and if they affect the template selection. An experienced musculoskeletal radiologist checked the quality of the dimension measurements. In total, 84 knee joint models for both imaging modalities were generated.

To find the closest FE model atlas for the subject, the measured dimensions were first normalized to the measured ML width values to have a comparison between the models with regards to shape.^32,37,40^ Then, the root mean square errors (RMSEs) were calculated between the normalized dimensions of the subject and FE models found in the atlas library. The best-matched FE model atlas was determined based on the minimum sum of the RMSE values, and this FE model atlas was selected to represent the subject (Fig. 1c). In this atlas-based approach, only medial compartment models were simulated to reduce computational burden and time. To obtain an even better representation of the subject's knee geometry, the selected FE medial compartment model was morphed to match the measured anatomical dimensions (Fig. 1d). The morphing was based on anisotropic scaling i.e., by multiplication of nodal coordinate (*x*, *y*, *z*) values of the best-matched atlas with the percentage difference in AP, ML and tibiofemoral JSW dimensions (d*x*, d*y* and d*z*) between the subject data and best-matched atlas (Fig. 1d).

### Loading and Boundary Conditions

The inputs for gait loading conditions were identical to our previous studies^31–33^ and are briefly explained here. Implemented loading conditions^6,23,24^ mimicked simplified walking (Fig. 1e). Loading consisted of the (1) flexion–extension rotation (flexion angle), (2) varus-valgus rotation, and (3) axial joint reaction force scaled with the BW of the subject.^33^ Flexion–extension and varus-valgus rotation were obtained from the best-matched template whereas axial joint reaction force scaled according to the subject’s BW (Fig. 1e) was considered with 50% of total joint forces in the medial compartment during gait.^23^ The medial compartment of the knee was implemented with the outputs obtained from the whole knee model. Following the approach in our previous study,^26^ the contribution of load transfer of medial meniscus was considered by subtraction from total reaction forces through the medial compartment model. In that study,^26^ it was demonstrated that at tibiofemoral contact, this simplification has a negligible effect on stress, fluid and contact pressure values.

The cartilage–bone interface of tibial cartilage was fixed, whereas the cartilage–bone interface of femoral cartilage was constrained to a reference point located between lateral and medial femoral epicondyles. These boundary conditions enabled us to control femoral motion with respect to the tibia by changing the loading conditions at the reference point. The effect of fluid flow was omitted in the medial meniscus as it was assumed that negligible fluid will flow during dynamic loading. Fluid flow through cartilage surfaces was restricted due to the assumption of negligible fluid flow in cartilage during dynamic loading (walking).^46^ More detailed information on boundary conditions and contact definitions can be found from our previous studies.^25,33^

More detailed information on boundary conditions and contact definitions can be found from our previous studies.^25,33^

### Material Properties and FE Simulations

The cartilage in the FE model was considered as a fibril reinforced poroviscoelastic (FRPVE) material involving a porous fluid-filled hyperelastic nonfibrillar matrix and a viscoelastic fibrillar matrix. Thus, the FRPVE material model can determine the contribution of the main tissue constituents (fluid, collagen and proteoglycans) on the biomechanical response of cartilage.^8,50^ The fibrillar matrix was modeled with 4 organized primary and 13 randomly oriented secondary fibrils.^8,29,50^ The total stress tensor of the material is of the form^22^:

1 $${{\varvec{\upsigma}}}^{{\text{t}}} = {{\varvec{\upsigma}}}_{{{\text{nf}}}} + \sum\limits_{i = 1}^{{{\text{tot}}\;{\text{f}}}} {{{\varvec{\upsigma}}}_{{\text{f}}}^{i} - p{\mathbf{I}}} ,$$

where the **σ**^t^ is the total stress tensor, **σ**_nf_ is the nonfibrillar matrix stress tensor, ${{\varvec{\upsigma}}}_{\mathrm{f}}^{\mathrm{i}}$ is the stress tensor of the *i*th fibril, tot f is the total number of fibrils, $\mathbf{I}$ is the unit tensor and *p* is the fluid pressure. Cartilage material properties were obtained from previous studies (Table 1).^15,25,30,32,33,47,50^ The FRPVE material model for cartilage tissue was implemented using user-defined material (UMAT) script in Abaqus. FE model simulations were performed in Abaqus/Standard (v6.18).

**Table 1:** FRPVE material parameters implemented in cartilage.^15,25,30,32,33,47,50^

FRPVE material parameter	Femoral cartilage	Tibial cartilage	Menisci
*E*_m_ (MPa)	0.215	0.106	–
*E*_0_ (MPa)	0.92	0.18	–
*E*_*ε*_ (MPa)	150	23.06	–
*ν*_m_ (–)	0.15	0.15	–
*η* (MPa s)	1062	1062	–
*k*_0_ (10^−15^ m^4^/N s)	6	18	–
*n*_f_	0.8–0.15*h*_*z*_	0.8–0.15*h*_*z*_	0.72
*E*_1_, *E*_2_ (MPa)	–	–	20
*E*_3_ (MPa)	–	–	159.6
*υ*_12_	–	–	0.3
*υ*_31_	–	–	0.78
*G*_13_ (MPa)	–	–	50

*E*_m_ nonfibrillar matrix modulus, *E*_0_ initial fibril network modulus, *E*_*ε*_ strain-dependent fibril network modulus, *ν*_m_ Poisson's ratio of the nonfibrillar matrix, *η* viscoelastic damping coefficient of fibrils, *k*_0_ initial permeability, *n*_f_ fluid fraction, *h*_*z*_ normalized depth, *E*_1_, *E*_2_, *E*_3_ radial, axial and circumferential Young’s moduli, respectively, *ν*_12_, *ν*_31_ Poisson’s ratios, *G*_13_ shear modulus

## FE Analyses

The following biomechanical cartilage responses were analyzed from the FE models: the maximum principal stress, maximum principal strain, minimum principal strain, collagen fibril strain, and fluid pressure. These mechanical parameters were determined as they are known to be associated with the load-bearing, degeneration and possible failure properties of cartilage.^7,22,49^ The mean (over cartilage–cartilage contact area) of the aforementioned parameters were obtained from the element centroids of the tibial cartilage as a function of the stance. Additionally, for determining the maximum values over stance, the element with the peak value of biomechanical parameter was identified. This value was averaged with the values of its connecting (neighboring) elements to eliminate abnormally high parameter values that originate from numerical contact discretization.

## Statistical Analyses

The normality of anatomical dimension measurement data was verified via the quantile–quantile plot, and the Shapiro–Wilk test. The anatomical dimensions obtained from both modalities were statistically compared using the parametric paired samples *t*-test with 95% confidence intervals (CIs). The significance level was *α* = 0.05. Correlation analysis of anatomical dimensions measured from both imaging modalities is presented separately in the Supplementary Material (S1, S2, S3, S4, S5). These analyses were conducted in IBM SPSS Statistics (v25, IBM Corporation, USA). Using MATLAB (2019b, The Mathworks, Inc., USA), we compared the 1-D trajectories of the averaged and peak values (over the contact area) of the biomechanical parameters from MRI- and X-ray-based models of each subject through the stance using the pairwise 1-D SPM,^36^ implemented via 1-D SPM toolbox (https://spm1d.org/). The normality of this data was tested via Shapiro–Wilk test prior to applying the parametric or nonparametric version of SPM. SPM method has an advantage over traditional zero-dimensional (0-D) parametric or nonparametric statistical tests (*t*-test or Wilcoxon signed-rank test) as it can consider various comparisons on smooth and random 1-D trajectories^36^ as in the case of the biomechanical FE model results.

## Results

### Comparison of Anatomical Dimensions Between X-Ray and MRI

There was no significant difference (*p* > 0.05) between the mean values of maximum medial (4.40 vs. 4.38 mm [95% CI for the difference − 0.06, 0.02]) and lateral (5.29 vs. 5.25 mm [95% CI − 0.002, 0.941]) JSWs between X-ray and MR images. On the other hand, the mean values of maximum AP dimensions of both medial (63.72 vs. 55.87 mm [95% CI 7.15, 8.54]) and lateral (66.43 vs. 63.72 mm [95% CI 2.24, 3.16]) elliptical-shaped condyles were greater in X-ray compared to MRI (*p* < 0.001). The mean value of the maximum ML distance of the distal femur was slightly greater (85.19 vs. 83.18 mm [95% CI − 2.37, − 1.66]) in MR than in X-ray images (*p* < 0.001). See Supplementary Table ST1 for the 95% CIs of measured parameters.

### Biomechanical Responses of X-Ray and MRI-Based FE Knee Model

**Figure 2:**
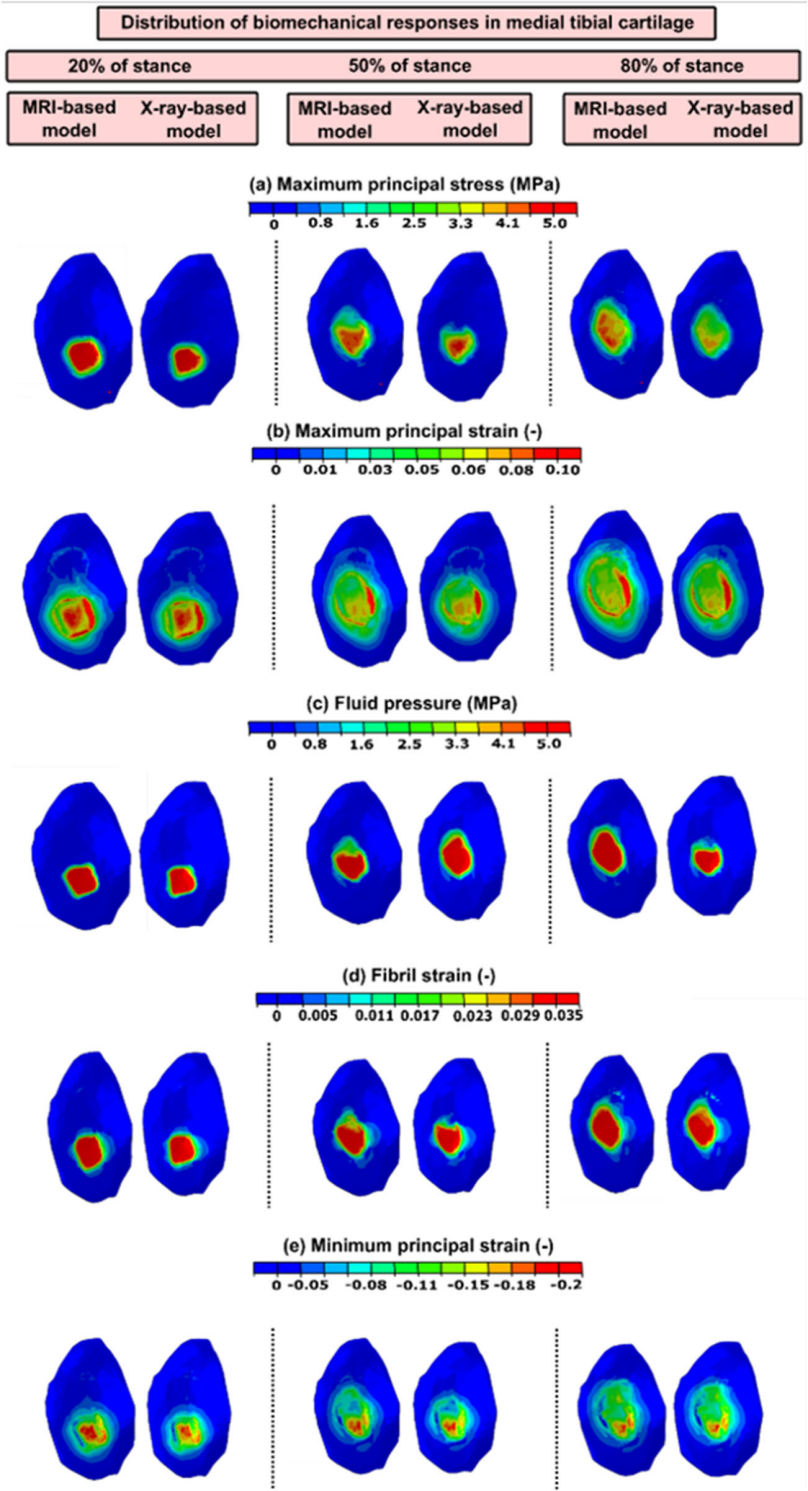
The simulated maximum principal stress and strain, fluid pressure, fibril strain and minimum principal strain distributions of a single subject in medial tibial cartilages of both MRI- and X-ray-based models are shown. These distributions of biomechanical responses were almost similar in both imaging modalities compartment models along 20, 50 and 80% of stance (same atlas source).

The maximum principal stress, maximum principal strain, fluid pressure, fibril strain and minimum principal strain distributions at the tibial plateau cartilage in MRI- and X-ray-based FE models of one subject (utilizing same atlas source) at 20, 50 and 80% of stance are shown in Fig. 2.

During the analyses, we observed that some of the model templates generated using radiographs matched with MRI generated templates (case 1, 24 models), but not in all models (case 2, 84 models), thus these cases are presented separately in the following sections.

**Case (1)** Average and peak biomechanical responses considering subjects (8) for whom the atlas-based approach determined the same best-matched templates from MRI and X-ray images (number of both MRI- and X-ray-based models = 24).

**Figure 3:**
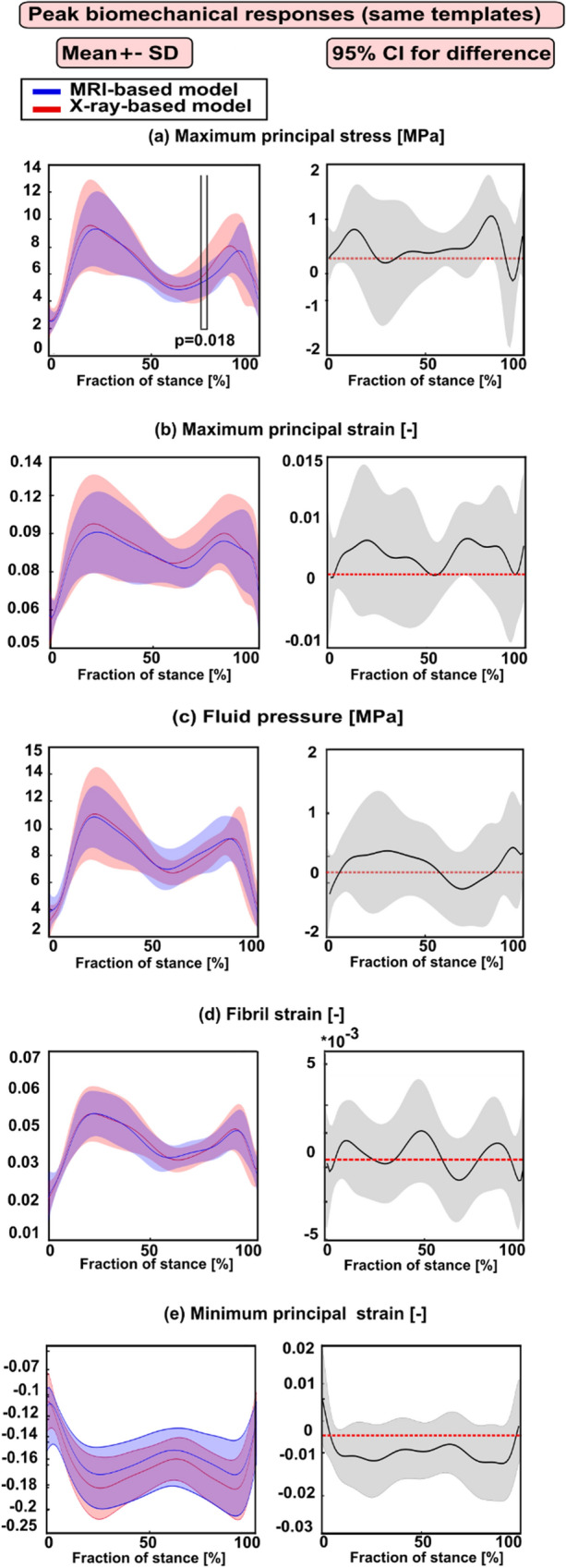
Left column: mean values (± standard deviation) for the peak biomechanical responses over the cartilage–cartilage contact region in the X-ray- and MRI-based FE knee models (with same templates) during the stance phase of the gait. (**a**) The maximum principal stress; (**b**) maximum principal strain; (**c**) fluid pressure; (**d**) fibril strain; and (**e**) minimum principal strain Statistical significances between the X-ray- and MRI-based models are shown based on the 1-D statistical parametric mapping. Right column: 95% confidence intervals (CI) for the difference (X-ray: MRI model) between the corresponding biomechanical response values during the stance phase of the gait.

**Figure 4:**
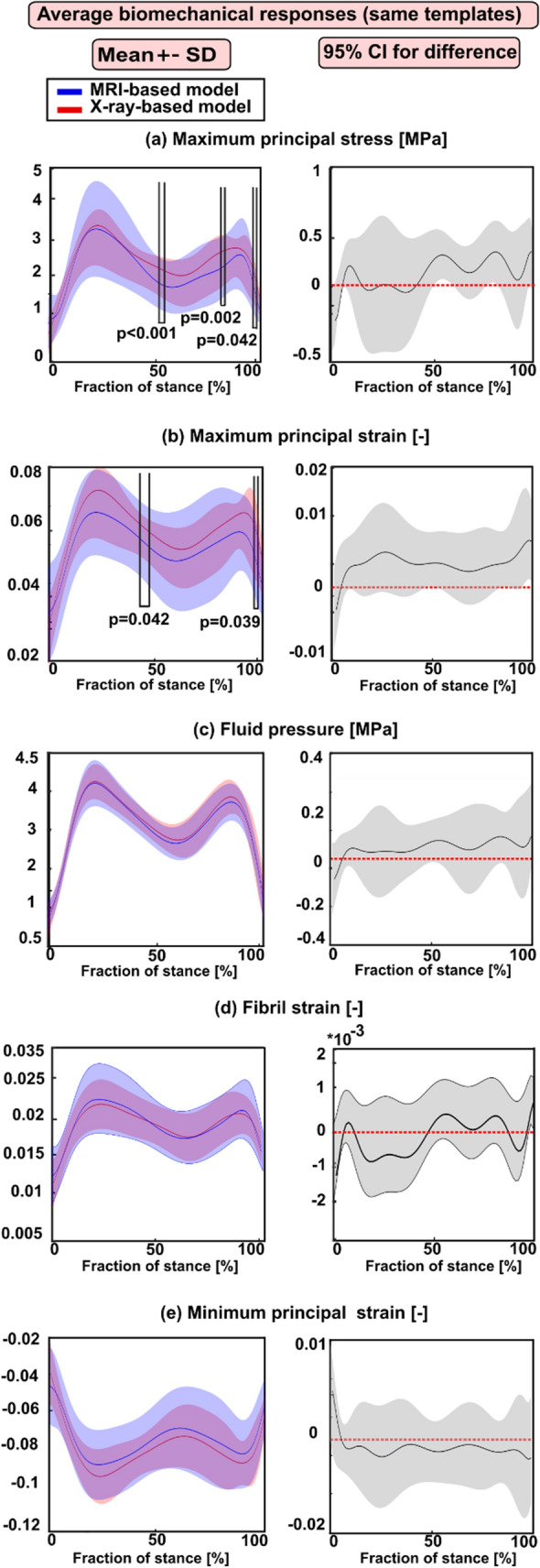
Left column: mean values (± standard deviation) for the averaged biomechanical responses over the cartilage–cartilage contact region in the X-ray- and MRI-based FE knee models (with same templates) during the stance phase of the gait. (**a**) The maximum principal stress; (**b**) maximum principal strain; (**c**) fluid pressure; (**d**) fibril strain; and (**e**) minimum principal strain Statistical significances between the X-ray- and MRI-based models are shown based on the 1D statistical parametric mapping. Right column: 95% confidence intervals (CI) for the difference (X-ray: MRI model) between the corresponding biomechanical response values during the stance phase of the gait.

In general, both peak and averaged biomechanical responses over the cartilage–cartilage contact region exhibited no statistical differences between the X-ray- and MRI-based FE models at the loading response and terminal stance phases of the gait cycle (Figs. 3 and 4). Yet, for maximum principal stress and strain, some differences were observed in very narrow timeframes during the stance.

**Case (2)** Average and peak biomechanical responses considering all subjects (28) for whom the atlas-based approach determined the same or different best-matched templates from MRI and X-ray images (number of both MRI- and X-ray-based models = 84).

**Figure 5:**
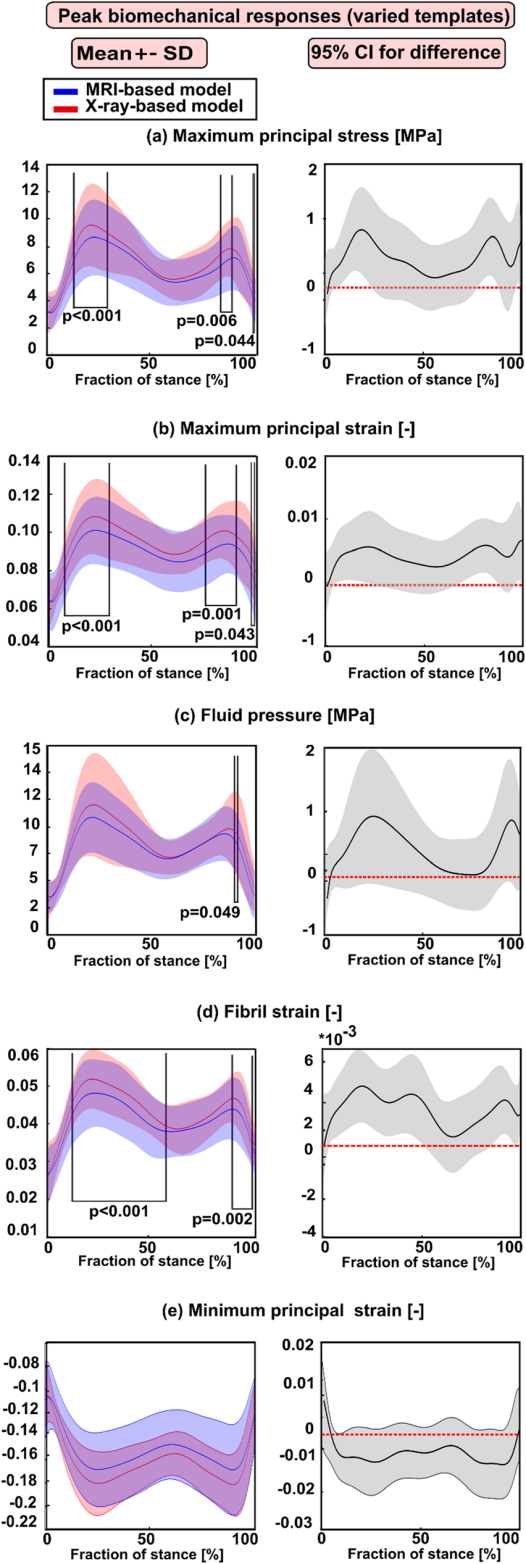
Left column: mean values (± standard deviation) for the peak biomechanical responses over the cartilage–cartilage contact region in the X-ray- and MRI-based FE knee models (with varied templates) during the stance phase of the gait. (**a**) The maximum principal stress; (**b**) maximum principal strain; (**c**) fluid pressure; (**d**) fibril strain; and (**e**) minimum principal strain Statistical significances between the X-ray- and MRI-based models are shown based on the 1-D statistical parametric mapping. Right column: 95% confidence intervals (CI) for the difference (X-ray: MRI model) between the corresponding biomechanical response values during the stance phase of the gait.

**Figure 6:**
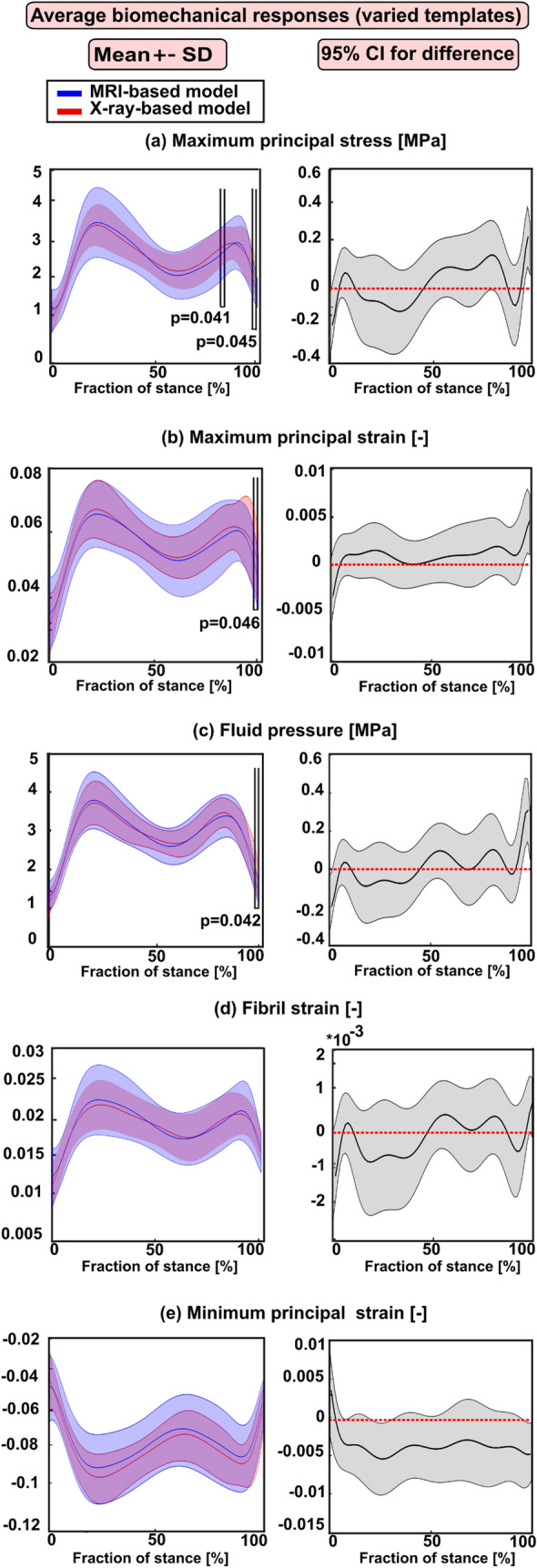
Left column: mean values (± standard deviation) for the averaged biomechanical responses over the cartilage–cartilage contact region in the X-ray- and MRI-based FE knee models (with varied templates) during the stance phase of the gait. (**a**) The maximum principal stress; (**b**) maximum principal strain; (**c**) fluid pressure; (**d**) fibril strain, and (**e**) minimum principal strain Statistical significances between the X-ray- and MRI-based models are shown based on the 1D statistical parametric mapping. Right column: 95% confidence intervals (CI) for the difference (X-ray: MRI model) between the corresponding biomechanical response values during the stance phase of the gait.

In general, the peak biomechanical responses over the cartilage–cartilage contact region exhibited statistical differences between the X-ray- and MRI-based FE models at the loading response and terminal stance phases of the gait cycle (Fig. 5) while the averaged biomechanical responses were statistically different but only in small timeframes at the end of the stance (Fig. 6).

## Discussion

In our study, we expanded our previously presented atlas-based FE knee joint modeling approach^30,32^ to incorporate knee radiographs for automated knee FE model generation and analysis of cartilage biomechanical responses. The model results were then compared with MRI-based models of the same subjects. Consistent with our hypothesis, we observed that peak and averaged cartilage stresses, strains and fluid pressures were not statistically different between the X-ray- and MRI-based models at loading response, terminal extension or along the majority of the stance when the same template was employed. However, the peak responses were statistically different along extended periods of stance between the X-ray- and MRI-based models that utilized varied templates.

Medial and lateral JSWs showed no significant difference between X-ray and MRI analyses. The maximum AP lengths of medial and lateral condyles were greater in X rays compared to MR images. Especially, the measured AP (medial) length was almost 8 mm greater in X rays than MRIs. Contrarily, the maximum ML length was slightly greater in MR images compared to that in X-ray images. Anatomical dimensions from both modalities were measured three times for each subject to evaluate the intra-rater reliability^14^ of the measurements and if their difference affect the template selection. Femoral condyles in radiographic images are visible in the lateral view. However, in this view, it becomes difficult to distinguish between both medial and lateral superimposed condyles (see Supplementary Figures S7, S8, S9, and S10). This causes uncertainties in the estimation of AP distance of femoral condyles from X-ray images (Table ST1) leading to sometimes different template selection among both modalities. Moreover, there was no knee positioning frame used when taking the lateral images. Therefore, there is variation in the flexion of the knee in the lateral images of X rays. As lateral views of MRIs and X rays are differently oriented, this may cause differences in condyles measurements from both imaging modalities. One of the limitations in this study was that the measurement accuracy of anatomical dimensions from sagittal and coronal views can be influenced by variations in internal-external positioning of the knee joint in X-ray images.^34^ Thus, in our presented biomechanical results obtained from X-ray- and MRI-based models, the only possible source of differences in mean and peak values of mechanical parameters arise presumably due to differences in anatomical dimensions and subsequent template selection for model generation. All other inputs in a model generation were the same in both approaches. Furthermore, different pixel size (MRI 0.6 × 0.6 mm^2^, X-ray 0.148 × 0.148 mm^2^) causes uncertainties in these analyses. These differences may be affected by X-ray image magnification which varies with patient positioning and size even with strict imaging protocols.

Statistical analysis focusing only on subjects (8) for whom the atlas-based approach determined the same best-matched templates based on all three anatomical dimensions from MRI and X-ray images showed that the peak responses were not statistically different at the loading response and terminal extension among both models (Fig. 3). However, when all subjects (28) were included in the analysis for whom the atlas-based approach determined sometimes the same and different templates, statistically meaningful differences (at loading response and terminal stance) in the peak values among both models were seen (Fig. 5). Despite the differences in dimensions and subsequent template selection, the X-ray- and MRI-based averaged modeling results were consistent with each other (Figs. 4 and 6). These results are quite encouraging as elevated levels of stresses and strains at the loading response after heel strike and terminal stance are mostly indicative of tissue failure or degeneration.^28^ The subject-specific (one subject) comparisons for the mean values of biomechanical are presented separately for each parameter in Supplementary Fig. S6. Mean values of biomechanical parameters were obtained by averaging over cartilage–cartilage contact area over the whole stance phase, while maximum values were obtained by averaging the biomechanical parameter values from the peak value element and its neighboring elements over the contact region during stance. A high stress or strain concentration even in one node can lead to overestimated peak results in certain periods of the stance phase. Taken together, the results emphasize that utilization of radiographs in knee FE modeling requires careful planning of knee X-ray imaging so that AP and ML dimension measurements would correspond to those of MRI.

All models were modeled with 8-node porous linear hexahedron C3D8P element type. Although mesh type was the same among all atlases, cartilage thickness and shape vary due to different knee joint sizes (see Supplementary Figs. S22 and S23). Hence, different atlases have different elements and nodes distribution. As a result, local stresses and strains may differ among different FE atlas models (Fig. S24). However, undesirable mesh collapsing due to secondary scaling was not the issue in this study. We observed that the relative difference between the greatest values of anatomical dimensions i.e., ML, AP medial, AP lateral, JSW medial and JSW lateral distances among the X-ray measurements and corresponding dimensions for atlases in the atlas-library was 0.4, 8, 5, 11 and 13% respectively. Similarly, the relative difference between the smallest values of anatomical dimensions i.e., ML, AP medial, AP lateral, JSW medial and JSW lateral distances among the X-ray measurements and corresponding dimensions for in atlas-library is 0.2, 7.9, 4, 8 and 10% respectively.

Atlas-based segmentation approaches are one of the most robust image-based segmentation techniques in the field of medical simulations, which perform classification and segmentation in one go,^42^ although deep learning-based techniques also look promising.^35^ In terms of generation of the model geometry, we compared earlier the results of the FE models obtained from the atlas-based approach and manually segmented knee joint models with experimental data.^32^ In that study, we showed that the models based on the atlas-based approach were able to predict the progression of OA similar to the models based on manual segmentation.

Biomechanical parameters like internal tissue strains have been linked with proteoglycan degeneration and subsequently fixed charge density (FCD) loss, while the collagen fibril strain and maximum principal stress have been suggested to be associated with collagen failure and degeneration.^2,20,33,44^ Specifically, the excessive minimum principal strain has been linked to PG loss via cell death.^41^ These mechanical parameters can be evaluated computationally along the gait from easily accessible X-ray imaging modality using our presented approach. The results were validated against MRI-based model responses (except peak responses in the case of varied templates selection). Yet, no prediction for OA progression was done in this study as there was no clinical follow-up information of disease progression for these subjects.^18^ However, it is important to note that our current methodology can be applied for the prediction of disease progression as our previously developed prediction algorithm^32^ is directly related to analyzed biomechanical parameters estimated in the current study. The algorithm was controlled by the accumulation of maximum principal stresses within cartilage during physiological loading which is suggested to reflect collagen failure in cartilage.^32^ The predictive FE model was generated from baseline (mainly KL0) and then it was used to predict OA progression, as seen experimentally by increased KL grades. To date, there have been no tools for evaluating cartilage degeneration and joint health, hence, the clinical applicability of the current methodology to predict cartilage failure and OA progression from X rays should be evaluated against a clinical follow-up study.

In the current study, we simulated biomechanical responses only from medial tibial cartilage and the lateral compartment of the knee was not considered in this study. The motivation for this was based on the literature as knee OA is known to occur particularly in the medial compartment of the tibiofemoral joint.^5^ This may be partially due to the characteristics of joint load distributions between lateral and medial compartments during walking.^32^ However, load distribution between lateral and medial compartments can affect the biomechanical responses and to tackle this we are currently working on adding tibiofemoral lateral compartment in our atlas-based workflow. Moreover, the addition of other knee structures like the patellofemoral compartment, including tendons, ligaments and muscles, will drastically increase computational time. To circumvent this, in the present study effects of these biological structures were included in the total force applied in the knee FE model. This can be justified since adding those tissues properly into the FE model needs information from subject-specific motion analysis data.

In our knee FE models, the subject-specific knee motion was not considered since the gait data was not collected from the patients. Furthermore, to reduce computational burden of simulations, the contribution of the medial meniscus was considered by subtracting the average contribution of meniscus from the total joint contact forces of the medial compartment. Hence, the current way to apply joint loading is not personalized. Nevertheless, it needs to be emphasized that the aim of this paper was not to produce subject-specific tissue responses during walking, but to investigate can this radiograph-based approach produce similar mechanical responses in cartilage as produced previously by the verified MRI-based approach.^32^ In that approach,^32^ MRI-based atlas knee models with generic gait loading were able to estimate volumetric cartilage degeneration which was indicative of OA progression. Moreover, clinicians widely use imaging modalities data to diagnose knee joint disorders and OA, whereas it is not viable to obtain motion data of every subject in the clinical setup. However, subject-specific motion data could further enhance and personalize our framework, hence, its role should be investigated.

We have currently only 21 atlases in our atlas library and we can simulate biomechanical parameters of only intact cartilages from this approach and cannot account for a plethora of knee cartilage pathologies such as cartilage lesions or defects. Hence, the inclusion of templates with cartilage lesions will lead to a more optimal representation of subject geometry. Additionally, only subjects with KL0 and KL1 grades were modeled. One of the reasons to include subjects based on KL0 and KL1 grade was to demonstrate that atlas-based approach^30^ is applicable for knee joints that don’t show X-ray-based cartilage degeneration.

When utilizing the same best-matched template,^32^ JSW has the most effective role on the predicted peak stress levels (see Supplementary Fig. S11), but among the models with the same and varied templates, peak responses are more sensitive to template selection. Due to statistical differences in anatomical dimensions among both imaging modalities (especially condyles and mediolateral distance of distal femur), the same template was not selected in all subjects. Different (but still best-matched based on the anatomical dimensions) templates produce different peak values (Fig. 5), which is logical as their shape and cartilage thicknesses in femoral and tibial cartilage are different. Correlation analysis between differences in anatomical dimensions and peak stresses and strains is given in Supplementary Material (Figs. S12, S13, S14, S15, S16, S17, S18, S19, S20 and S21). Possible ways to improve our current methodology are to utilize different parametrization of the knee shape (e.g., using ellipsoidal fits of condyles) and subject characteristics parameters such as age, gender, height, the weight of the subject when selecting the best-matched template.

To conclude, the results of the study suggest that peak and averaged biomechanical responses were not statistically different between MRI- vs. X-ray-derived FE models only when the same atlas-template was selected. However, if a different template was selected, peak biomechanical responses in the X-ray-based models were statistically greater compared to MRI-based models along the stance, yet, further studies are required to clarify whether this difference is clinically meaningful. Interestingly, the average responses remained statistically not different between MRI- vs. X-ray-based models even with the utilization of different templates. Thus, the presented workflow may enable X-ray imaging-based computational modeling for evaluating knee joint biomechanics.

## Supplementary Information

Below is the link to the electronic supplementary material.

Supplementary file1 (PDF 2440 kb)
