# Correction to: Rapid X-Ray-Based 3-D Finite Element Modeling of Medial Knee Joint Cartilage Biomechanics During Walking

**DOI:** 10.1007/s10439-022-02957-6

**Published:** 2022-03-24

**Authors:** Sana Jahangir, Ali Mohammadi, Mika E. Mononen, Jukka Hirvasniemi, Juha-Sampo Suomalainen, Simo Saarakkala, Rami K. Korhonen, Petri Tanska

**Affiliations:** 1 Department of Applied Physics, University of Eastern Finland, POB 1627, 70211 Kuopio, Finland; 2 Department of Radiology & Nuclear Medicine, Erasmus MC University Medical Center, Rotterdam, The Netherlands; 3 Diagnostic Imaging Center, Kuopio University Hospital, Kuopio, Finland; 4 Research Unit of Medical Imaging, Physics and Technology, Faculty of Medicine, University of Oulu, Oulu, Finland; 5 Department of Diagnostic Radiology, Oulu University Hospital, Oulu, Finland

## Correction to: Annals of Biomedical Engineering 10.1007/s10439-022-02941-0

The Acknowledgments paragraph should be replaced by the following funding paragraph:

Funding is provided by the Academy of Finland (#324529, #324994, #328920, #334773—under the Frame of ERA PerMed), the Strategic Funding of University of Eastern Finland, Sigrid Juselius Foundation and Alfred Kordelin Foundation (#190317). Open access funding provided by University of Eastern Finland (UEF) including Kuopio University Hospital.

Also, the ORCID ID for author Petri Tanska is http://orcid.org/0000-0002-9684-6902.

