# Supplementary material for: Rapid X-Ray-Based 3-D Finite Element Modeling of Medial Knee Joint Cartilage Biomechanics During Walking
Source: Ann Biomed Eng. 2022 Mar 9;50(6):666–79. doi: 10.1007/s10439-022-02941-0 (PMC9079039; doi:10.1007/s10439-022-02941-0)
Supplement: Supplementary file 1 — Supplementary file1 (PDF 2440 kb) [file 10439_2022_2941_MOESM1_ESM.pdf]

Supplementary material for:

Rapid X-ray-based 3-D finite element modeling of medial knee joint cartilage biomechanics during walking

Sana Jahangir[1], Ali Mohammadi[1], Mika E. Mononen[1], Jukka Hirvasniemi[2], Juha-Sampo Suomalainen[3], Simo Saarakkala[4,5], Rami K. Korhonen[1], Petri Tanska[1]

[1] Department of Applied Physics, University of Eastern Finland, Kuopio, Finland

[2] Department of Radiology & Nuclear Medicine, Erasmus MC University Medical Center, Rotterdam, the Netherlands

[3] Diagnostic Imaging Center, Kuopio University Hospital, Kuopio, Finland

[4] Research Unit of Medical Imaging, Physics and Technology, Faculty of Medicine, University of Oulu, Oulu, Finland

[5] Department of Diagnostic Radiology, Oulu University Hospital, Oulu, Finland

***Corresponding Author:**

Sana Jahangir, M.Sc.

Address: POB 1627, FI-70211, Kuopio, Finland

Telephone: +358 505738151

Email: sana.jahangir@uef.fi

STATISTICAL ANALYSES

A pearson's correlation coefficient was computed to assess the relationship between each anatomical dimension (including tibiofemoral joint space width (JSW) from both the medial and lateral compartments (JSW medial and JSW lateral), maximum anterior-posterior (AP) lengths from the medial and lateral condyles (AP medial and AP lateral) of distal femur, and the maximum medial-lateral (ML) width of distal femur) measured from both MR and X-ray images. Correlation is significant at 0.05 level. Correlation analysis is presented in Figures S1, S2, S3, S4, S5.

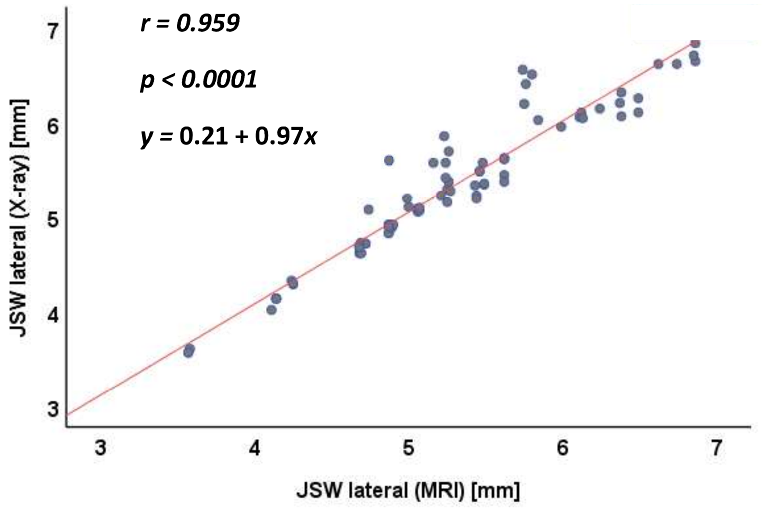

Figure S1: Pearson's correlation between JSW lateral measured from both MR and X-ray images (n = 84).

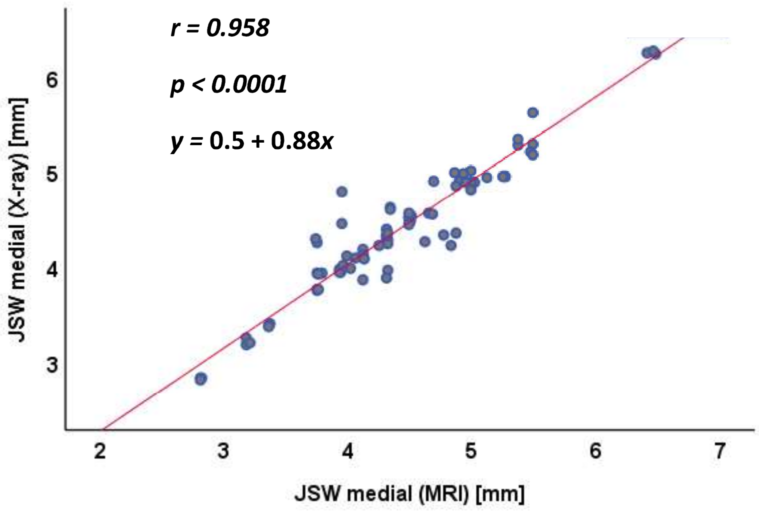

Figure S2: Pearson's correlation between JSW medial measured from both MR and X-ray images (n = 84).

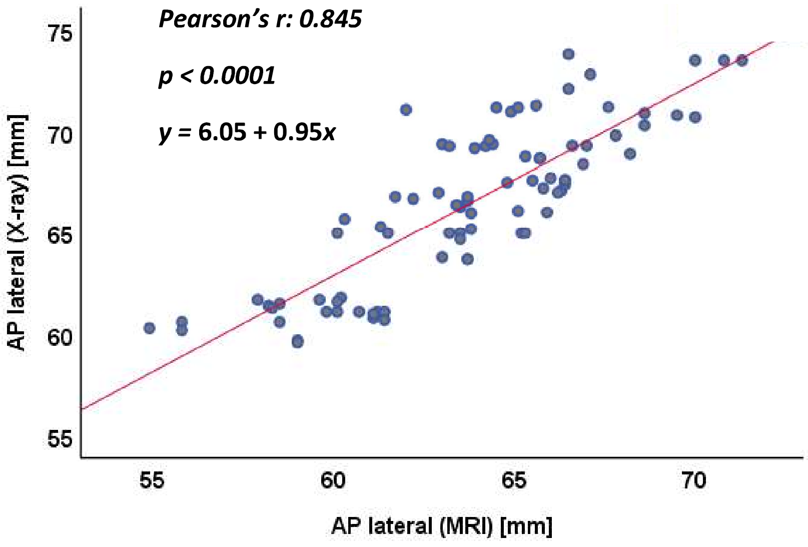

Figure S3: Pearson's correlation between AP lateral measured from both MR and X-ray images (n = 84).

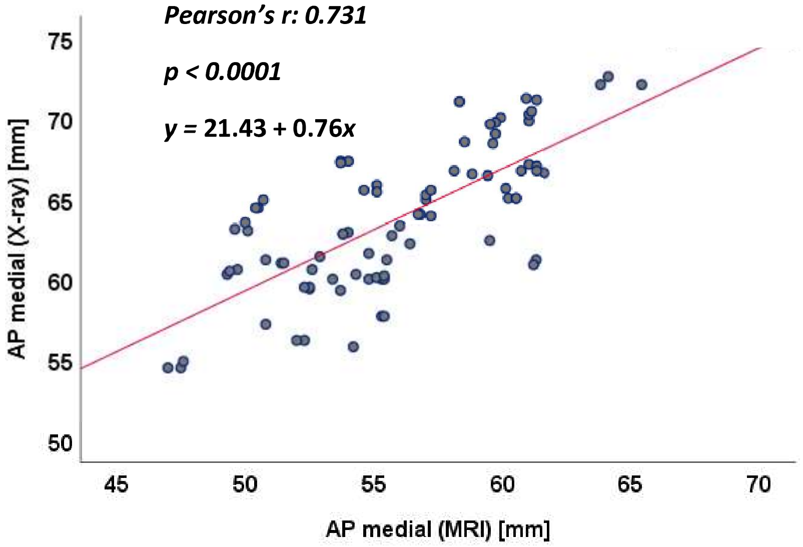

Figure S4: Pearson's correlation between AP medial measured from both MR and X-ray images (n = 84).

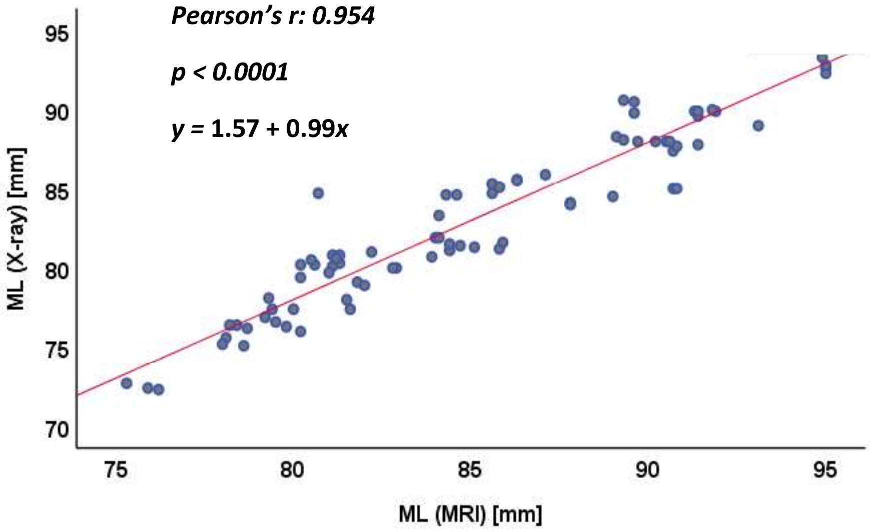

Figure S5: Pearson's correlation between ML measured from both MR and X-ray images (n = 84).

Results

Table ST1:

Mean, 95% confidence interval (CI) of mean and 95% confidence interval (CI) of the difference (X-ray - MRI) for the maximum medial-lateral distance (ML), maximum anterior-posterior length of the medial and lateral condyles (AP), and maximum joint space width (JSW) in the medial and lateral condyles obtained through quantifying the anatomical measurements of the knee in MRI and X-ray imaging.

**Anatomical dimension**	**Mean MRI, [95% CI]**	**Mean X-ray, [95% CI]**	**95% CI of the difference (X-ray-MRI)**	***p*-value**
ML (mm)	85.19, [84.06, 86.33]	83.18, [81.99, 84.36]	[-2.37, -1.66]	<0.001
AP medial (mm)	55.87, [54.94, 56.79]	63.72, [62.76, 64.68]	[7.15, 8.54]	<0.001
AP lateral (mm)	63.72, [62.96, 64.48]	66.43, [65.57, 67.28]	[2.24, 3.16]	<0.001
JSW medial (mm)	4.38, [4.24, 4.56]	4.40, [4.23, 4.53]	[-0.06, 0.02]	0.389
JSW lateral (mm)	5.25, [5.08, 5.42]	5.29, [5.12, 5.46]	[-0.002, 0.941]	0.061

Biomechanical responses of X-Ray and MRI-based FE knee model (one subject)

The subject-specific (one subject) comparisons for the mean values of maximum principal stress, maximum principal strain, minimum principal strain, fluid pressure, fibril strain and minimum principal strain are presented separately for each parameter in Figure S6.

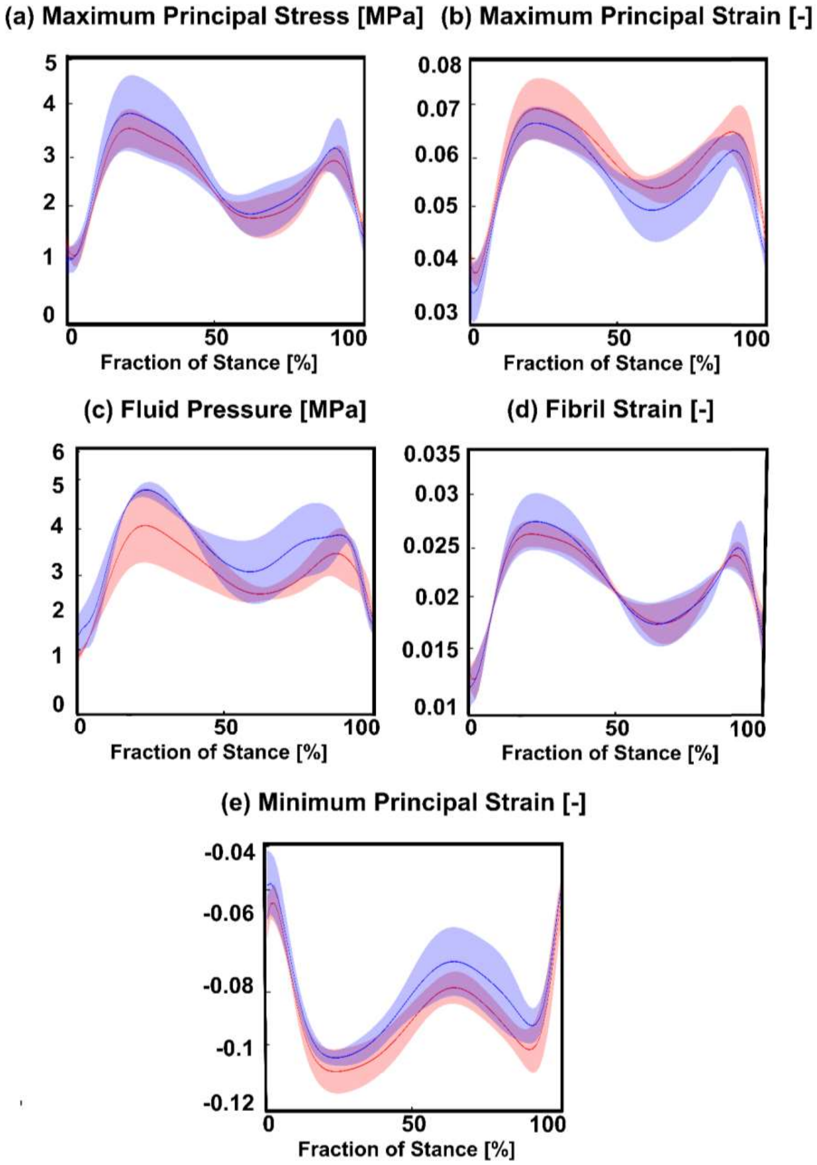

Figure S6: The subject-specific (one subject) comparison for the mean values of maximum principal stress, maximum principal strain, fluid pressure, fibril strain and minimum principal are presented. Mean values of the biomechanical parameters at the contact area are predicted by three X-ray- and MRI-based models for one subject. The solid line represents the mean value from three trials, and the shaded area represents the standard deviation.

Measurement of maximum anterior-posterior length of medial and lateral condyles from X-ray images

Case 1: Fully superimposed condyles

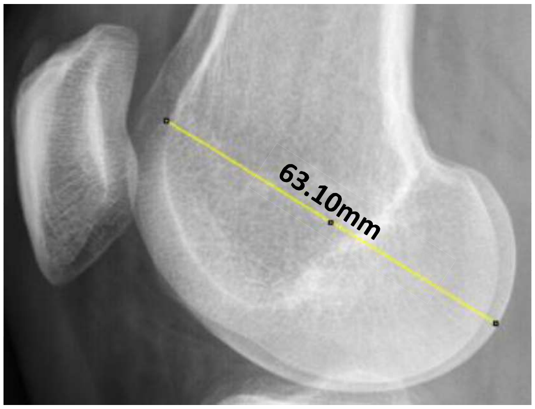

Figure S7: Maximum anterior-posterior length of medial condyle

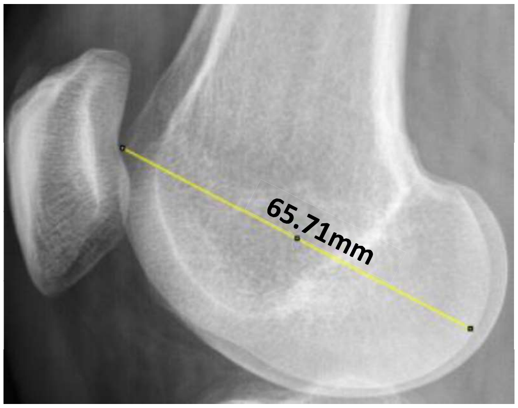

Figure S8: Maximum anterior-posterior length of lateral condyle

Case 2: Not fully superimposed condyles

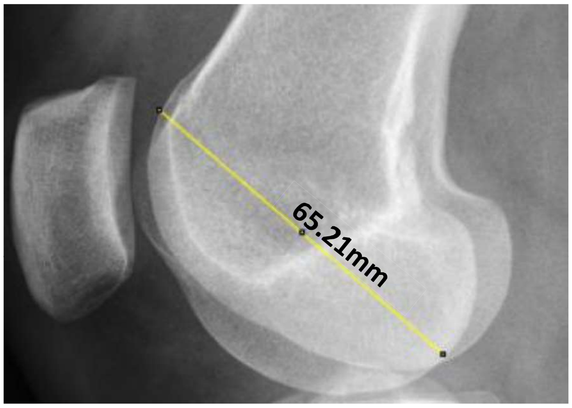

Figure S9: Maximum anterior-posterior length of medial condyle

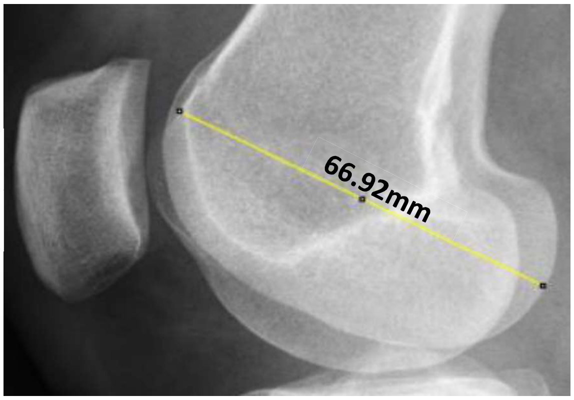

Figure S10: Maximum anterior-posterior length of lateral condyle

Correlation analysis between anatomical dimensions and peak values of maximum principal stress from X-ray-based models (one template)

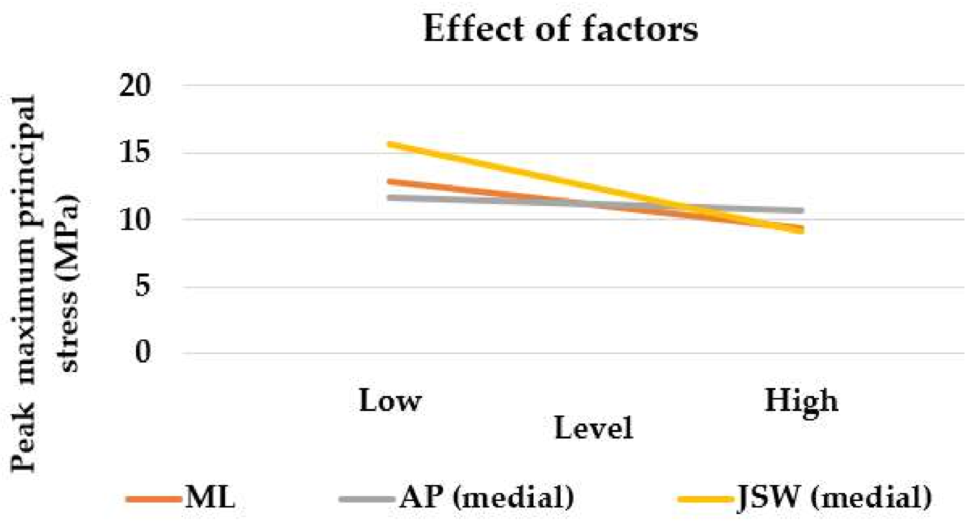

Factors	Level	
	Low	High
ML [mm]	76.01	94.40
AP (medial) [mm]	54.50	72.60
JSW (medial) [mm]	3.25	4.90

Figure S11: Means of factorial design with three factors and two levels analysis is given. Low and high levels are minimum and maximum values of mediolateral distance (ML), anterior-posterior length and joint space width measured at medial compartment from X-ray images. For this factorial analysis X-ray-based models with same template were selected. In this analysis of means of factorial design, there are three factors i.e, ML, AP and JSW distances from X-rays which have two levels i.e, minimum and maximum values of these measurements. The line graph shows that main effect of JSW leads to higher estimated peak stress levels. A main effect is the effect of one independent variable (e.g., JSW) on the dependent variable (peak stress levels)—averaging across the levels of the other independent variable. Hence, JSW has the most effective role on the predicted peak stress levels in case of same template utilization in models.

Correlation analysis between difference in anatomical dimensions and peak values of maximum principal stress and strain at the loading response of stance (multiple templates)

Pearson's and spearman's correlation coefficients were computed to assess the relationship between difference among anatomical dimension measured from both X-ray and MRI images and difference in peak values of maximum principal stress and strain at first peak of stance among X-ray- and MRI-based models respectively. Correlation is significant at $p < 0.05$. Correlation analysis is presented in Figures S12-S21. Differences in peak stresses and AP lateral dimension (X-ray vs MRI) were correlated ($p = 0.017$, Pearson's $r = 0.260$). Similarly, differences in peak strains and AP lateral were correlated ($p = 0.002$, Spearman's $r = 0.332$).

Correlation analysis between differences in anatomical dimensions and peak values of maximum principal stress at the loading response of stance (X-ray- vs. MRI-based models):

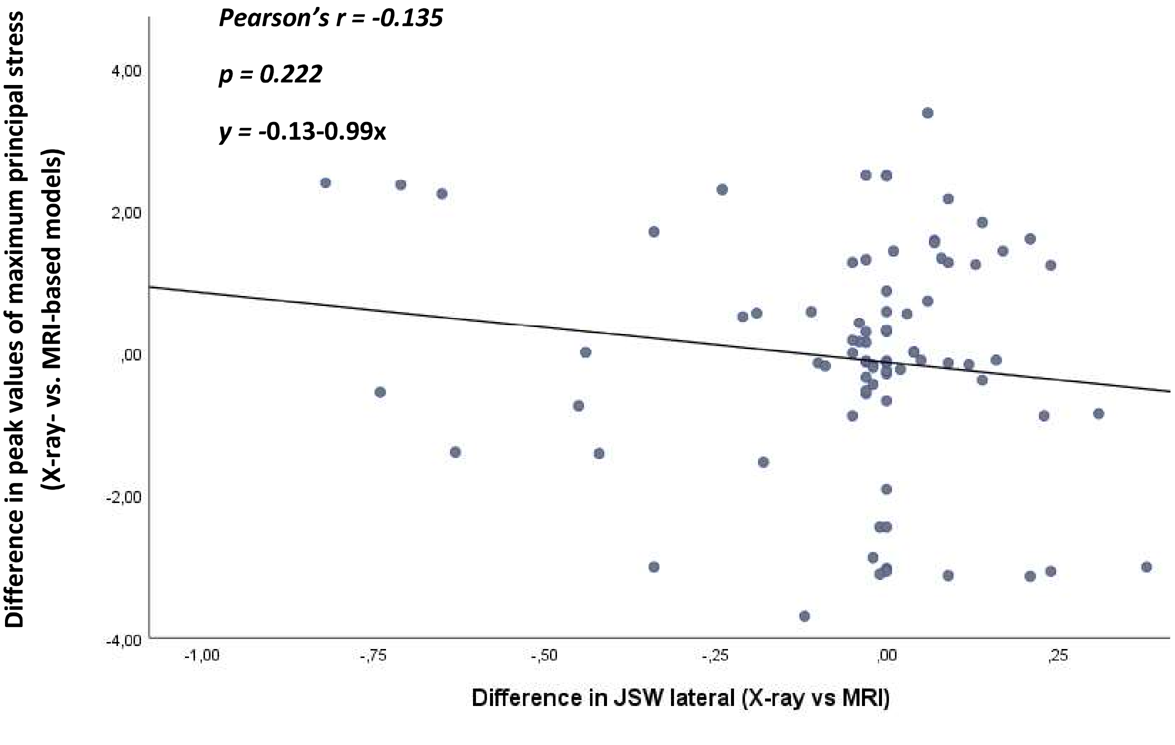

Figure S12: *Pearson's* correlation between difference in JSW lateral measurements (X-ray vs MRI) and peak values of maximum principal stress among X-ray vs. MRI-based models ($n = 84$).

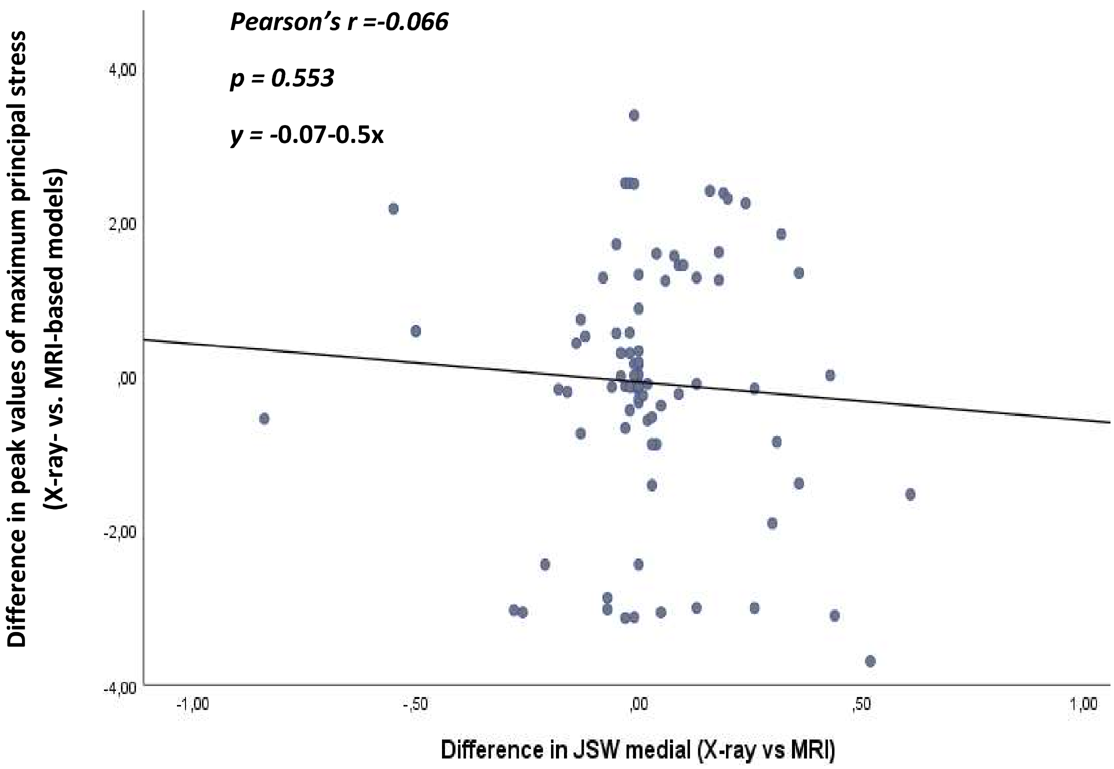

Figure S13: *Pearson's* correlation between difference in JSW medial measurements (X-ray vs MRI) and peak values of maximum principal stress among X-ray vs. MRI-based models (*n* = 84).

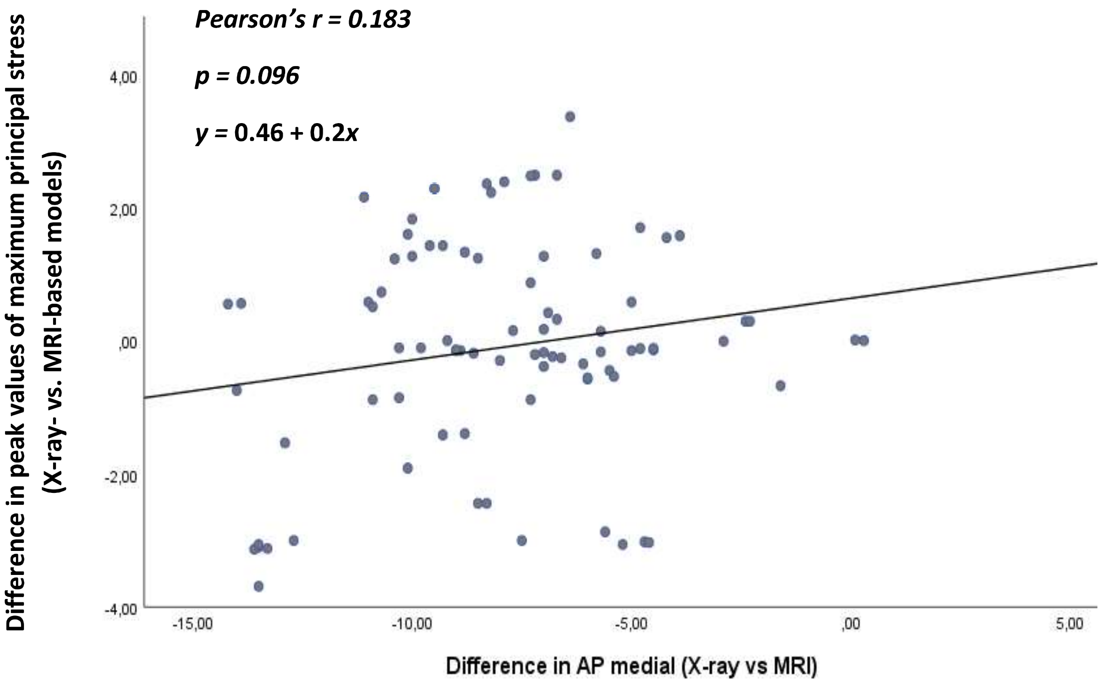

Figure S14: *Pearson's* correlation between difference in AP medial measurements (X-ray vs MRI) and peak values of maximum principal stress among X-ray vs. MRI-based models (*n* = 84).

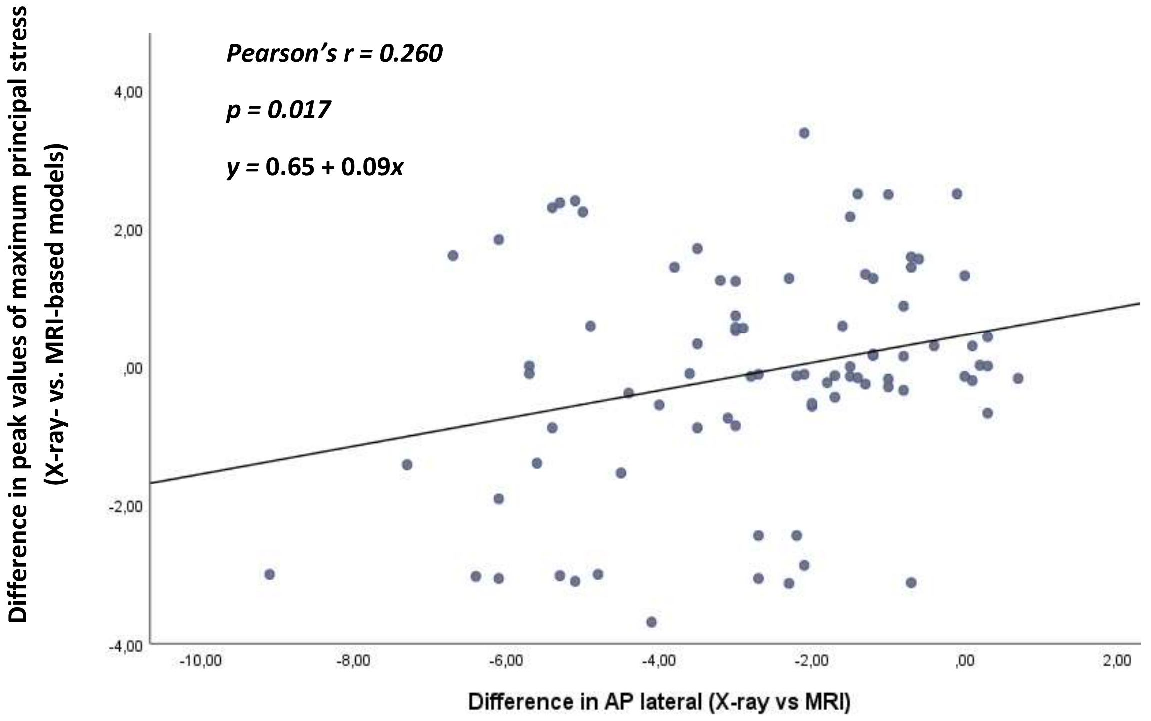

Figure S15: *Pearson's* correlation between difference in AP lateral measurements (X-ray vs MRI) and peak values of maximum principal stress among X-ray vs. MRI-based models (*n* = 84).

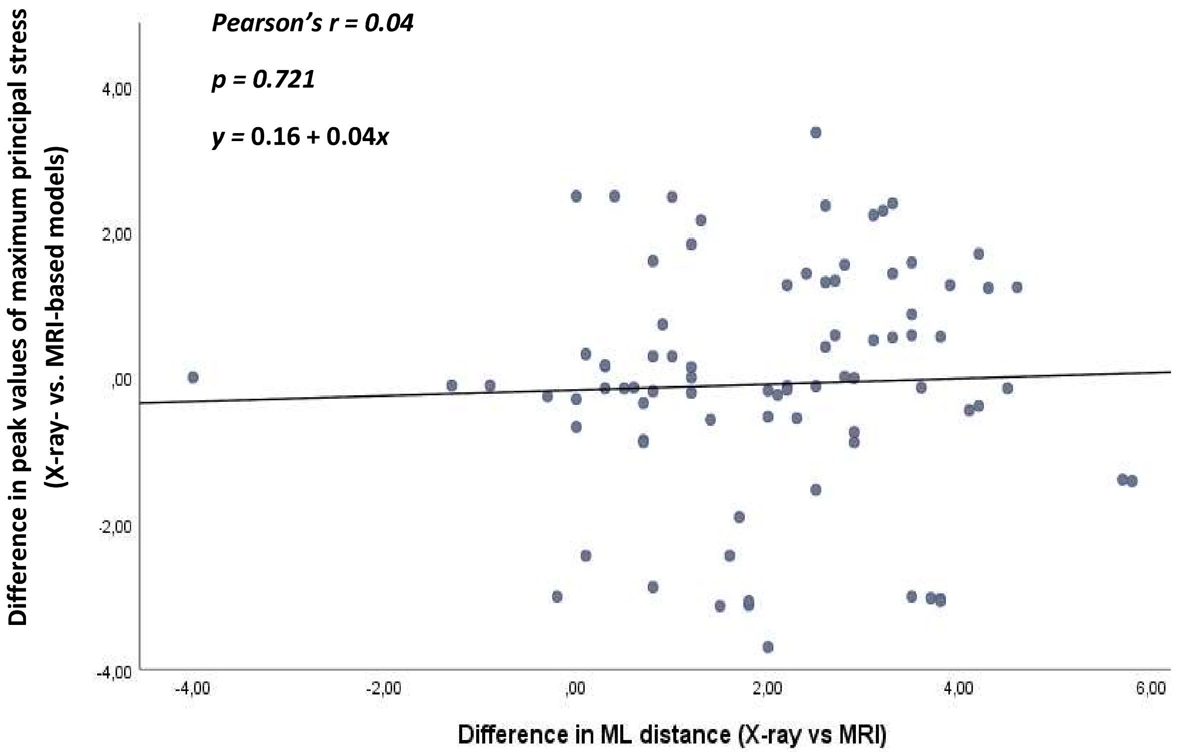

Figure S16: *Pearson's* correlation between difference in ML measurements (X-ray vs MRI) and peak values of maximum principal stress among X-ray vs. MRI-based models (*n* = 84).

Correlation analysis between differences in anatomical dimensions and peak values of maximum principal strain at the loading response of stance (X-ray- vs. MRI-based models)

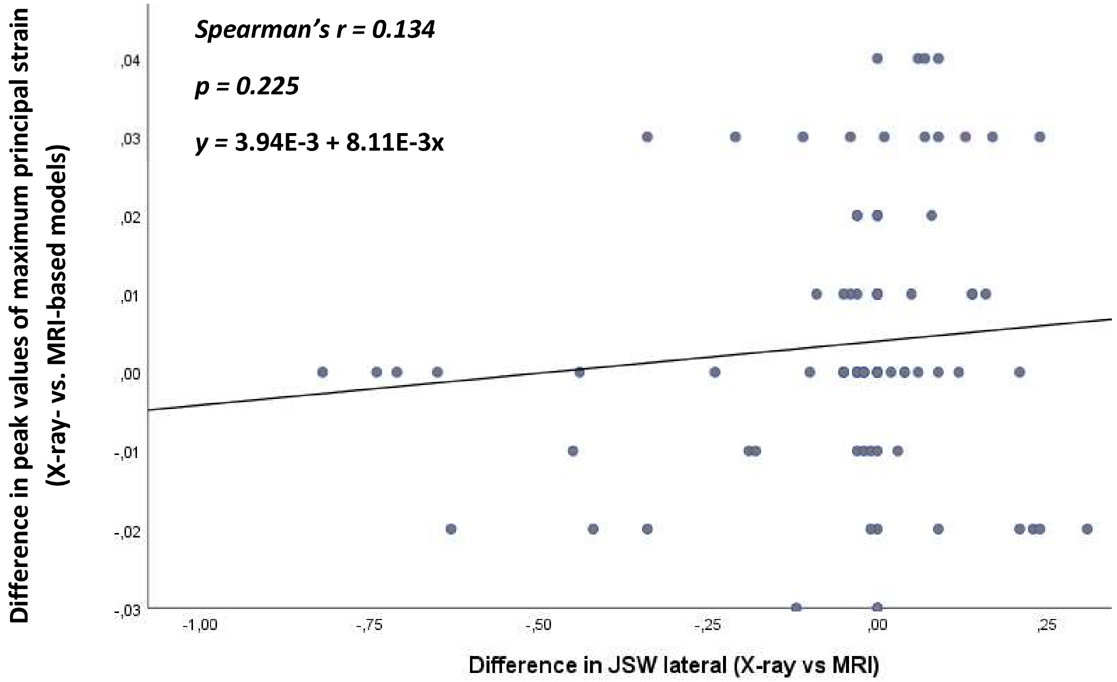

Figure S17 *Spearman's* correlation between difference in JSW lateral measurements (X-ray vs MRI) and peak values of maximum principal strain among X-ray vs. MRI-based models (n = 84).

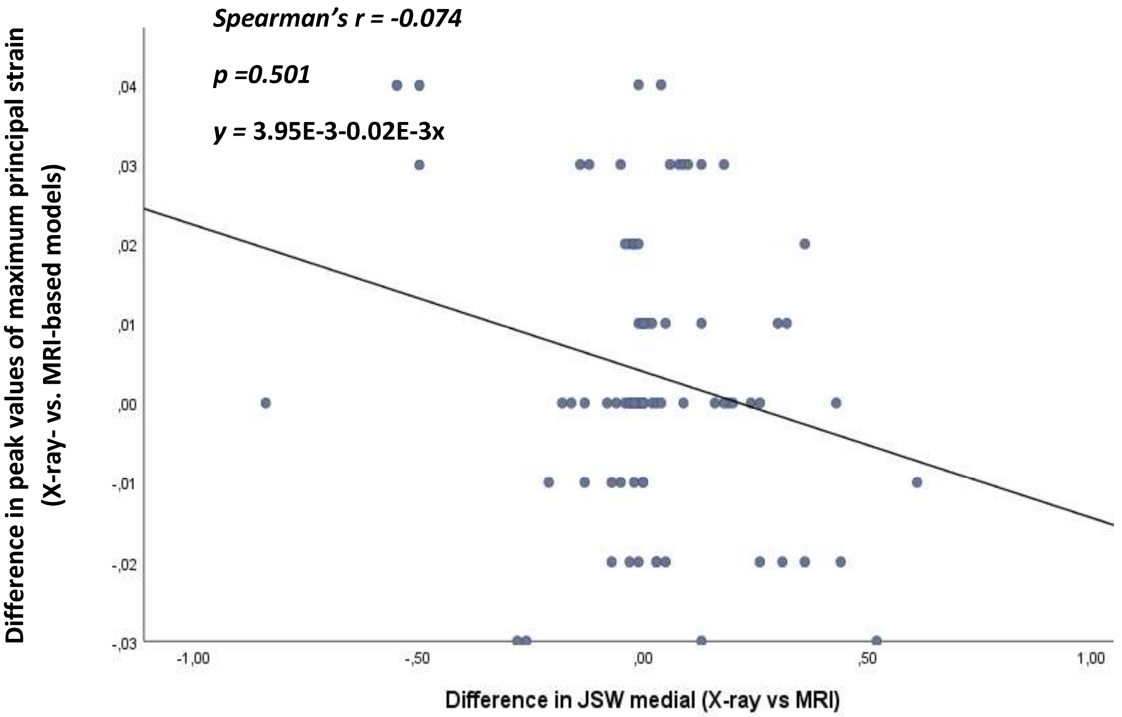

Figure S18: *Spearman's* correlation between difference in JSW medial measurements (X-ray vs MRI) and peak values of maximum principal strain among X-ray vs. MRI-based models (n = 84).

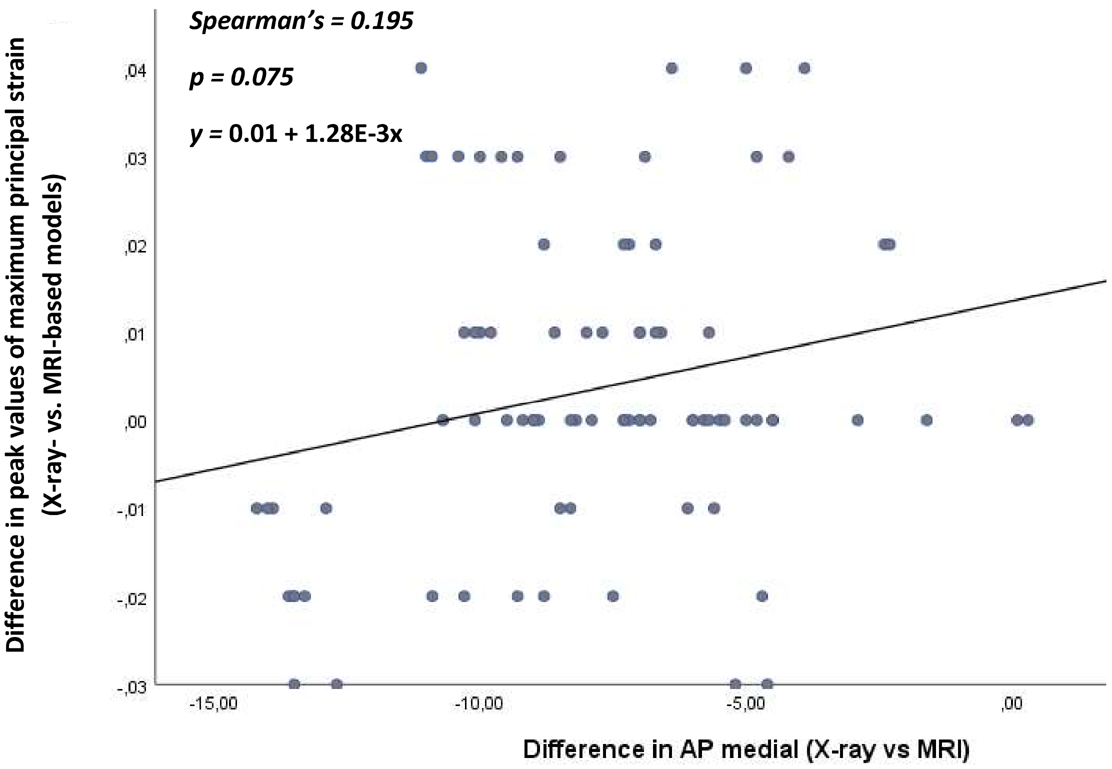

Figure S19: *Spearman's* correlation between difference in AP medial measurements (X-ray vs MRI) and peak values of maximum principal strain among X-ray vs. MRI-based models (*n* = 84).

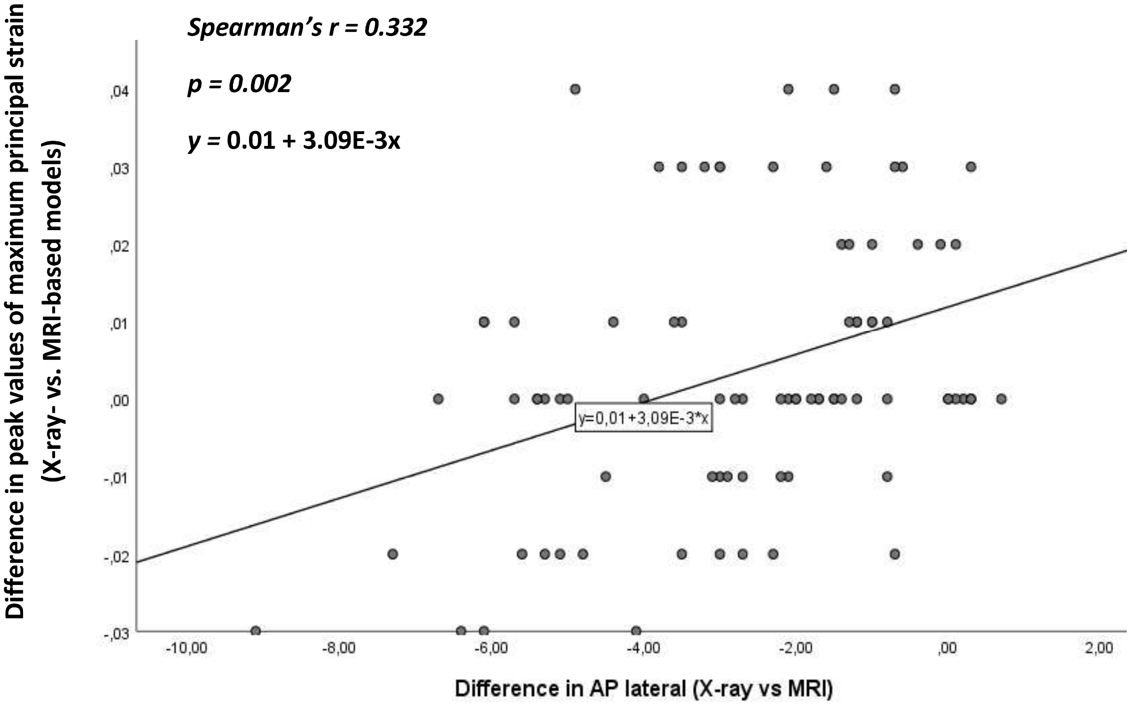

Figure S20: *Spearman's* correlation between difference in AP lateral measurements (X-ray vs MRI) and peak values of maximum principal strain among X-ray vs. MRI-based models (*n* = 84).

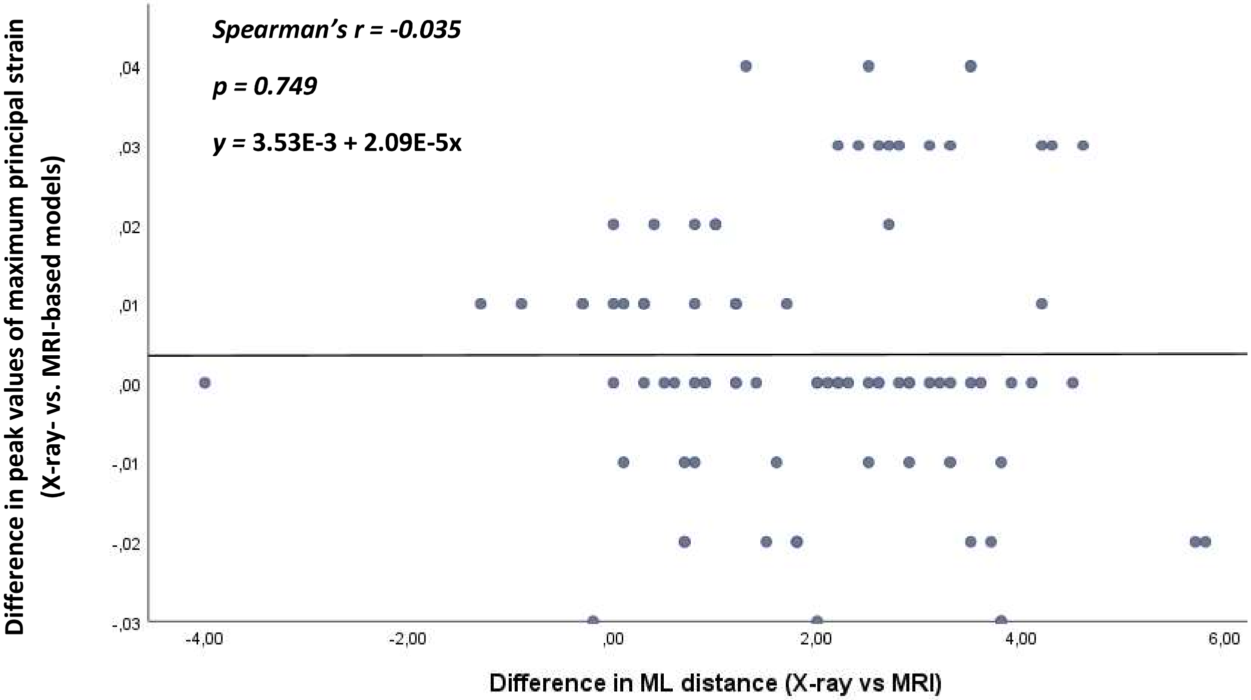

Figure S21: *Spearman's* correlation between difference in ML measurements (X-ray vs MRI) and peak values of maximum principal strain among X-ray vs. MRI-based models (*n* = 84).

Visualization of FE meshes in X-ray versus MRI-based models of same subject (with different best-matched templates)y

Although mesh type was same among all atlases, cartilage thickness and shape vary due to different knee joint sizes (Supplementary Figures S22-S23). Hence, different atlases have different elements and nodes distribution.

MRI-based model (surface and internal mesh):

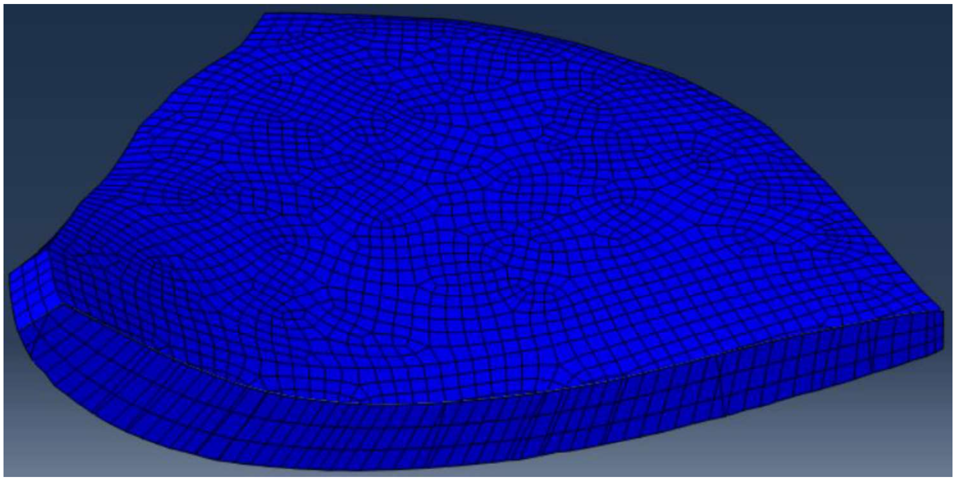

X-ray-based model (surface and internal mesh):

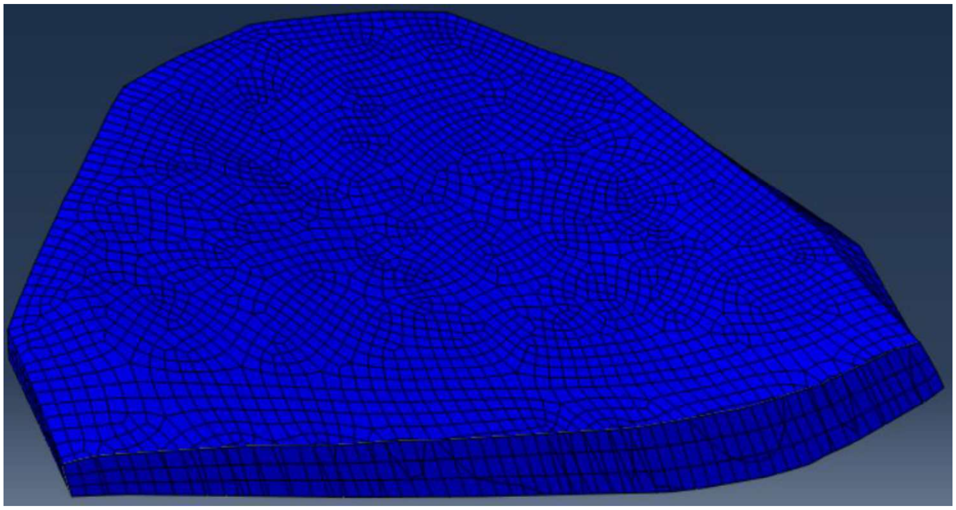

Figure S22: Visualization of meshes (surface and internal mesh) of X-ray versus MRI-based models of same subject (with different best-matched templates).

Visualization of FE meshes in X-ray versus MRI-based models of same subject (with same best-matched templates)

Elements and nodes distribution is identical in same best-matched templates.

MRI-based model (surface and internal mesh):

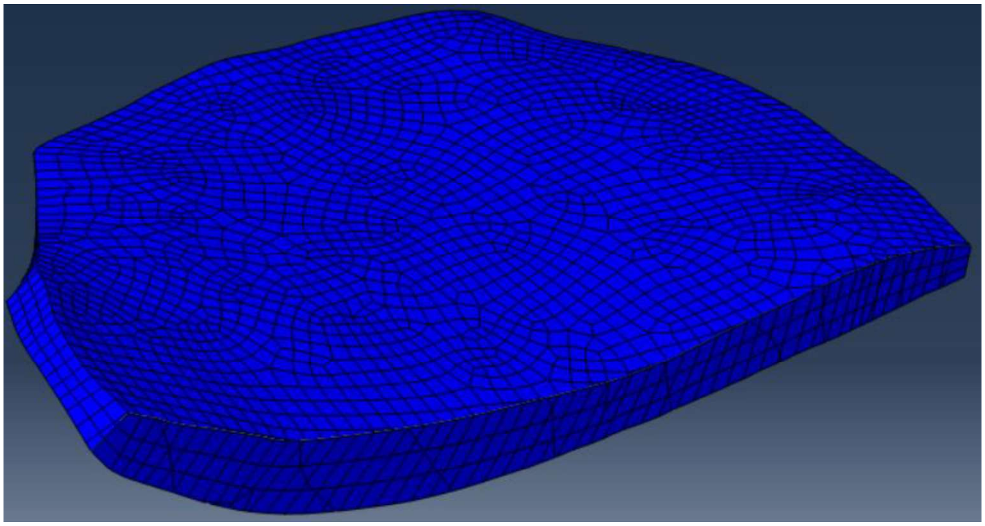

X-ray-based model (surface and internal mesh):

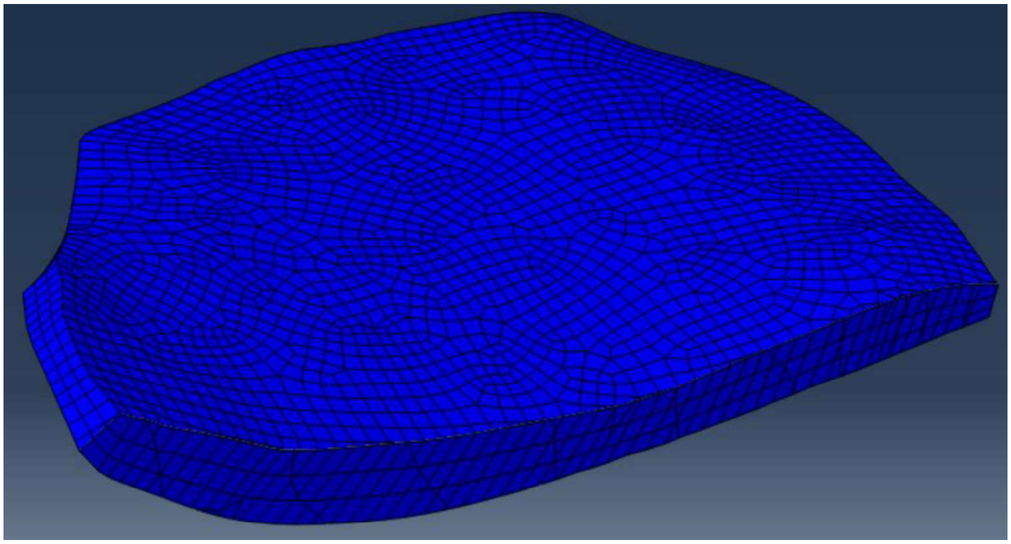

Figure S23: Visualization of meshes (surface and internal mesh) of X-ray versus MRI-based models of same subject (with same best-matched templates).

Distribution of maximum principal stress in medial tibial cartilage of MRI- vs X-ray-based models of same subject (with different best-matched template)

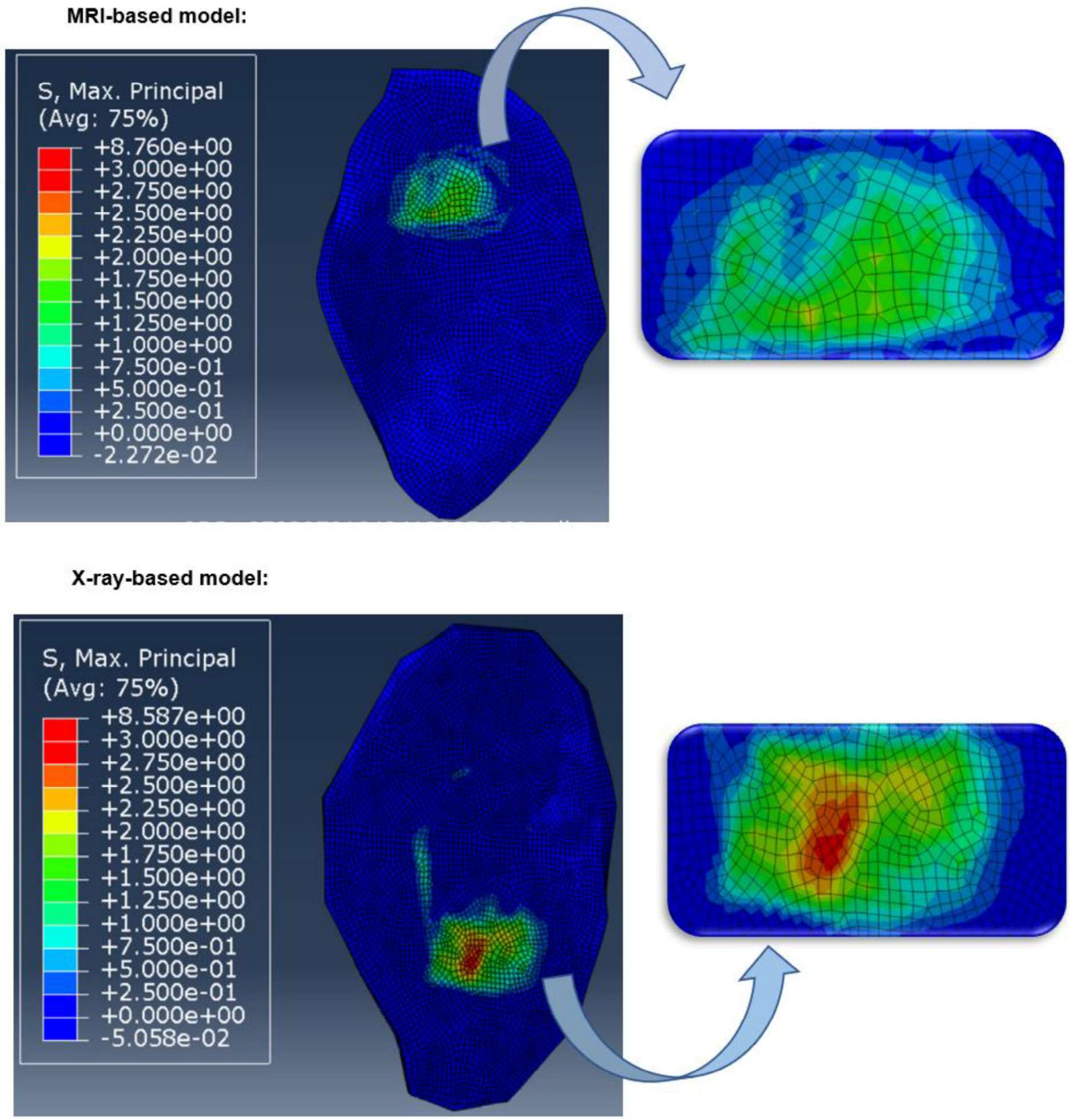

Figure S24: Distribution of maximum principal stress in medial tibial cartilage of MRI- vs X-ray-based models of same subject (with different best-matched template).
